# Achieving robust labeling above the circle of Willis with vessel‐encoded arterial spin labeling

**DOI:** 10.1002/mrm.30542

**Published:** 2025-07-01

**Authors:** Hongwei Li, Yang Ji, He Wang, Zhensen Chen, Tiansheng Qian, Yujun Liao, Jian Wang, Joseph G. Woods, Yuriko Suzuki, Thomas W. Okell

**Affiliations:** ^1^ Institute of Science and Technology for Brain‐inspired Intelligence Fudan University Shanghai People's Republic of China; ^2^ Wellcome Centre for Integrative Neuroimaging, FMRIB Division, Nuffield Department of Clinical Neurosciences University of Oxford Oxford UK; ^3^ Department of Electronic Engineering and Information Science, School of Information Science and Technology University of Science and Technology of China Hefei People's Republic of China; ^4^ MOE Key Laboratory of Computational Neuroscience and Brain‐Inspired Intelligence Fudan University Shanghai People's Republic of China; ^5^ Department of Neurosurgery Fudan University Huashan Hospital Shanghai People's Republic of China; ^6^ Clinical Medical Center of Neurosurgery Shanghai Key Laboratory of Brain Function and Restoration and Neural Regeneration Shanghai People's Republic of China; ^7^ Department of Neurosurgery, The Second People's Hospital of Changzhou The Third Affiliated Hospital of Nanjing Medical University, Changzhou Medical Center Changzhou People's Republic of China

**Keywords:** encoding scheme, SNR efficiency, vascular territory, vessel‐encoded arterial spin labeling (VEASL), vessel‐selective

## Abstract

**Purpose:**

To improve the robustness of noninvasive vessel‐selective perfusion imaging and angiography using vessel‐encoded arterial spin labeling (VEASL) when applied to complex vascular geometries, such as above the circle of Willis (CoW) in the brain.

**Methods:**

Our proposed improved optimized encoding scheme (IOES) better accounts for vascular geometry and the VEASL encoding process, leading to more SNR‐efficient encodings than previous approaches. Pseudo‐continuous arterial spin labeling (PCASL) parameters were optimized for a thinner labeling region, allowing tortuous vessels to be more accurately treated as single points within the labeling plane. Our optimized approach was compared to the original OES method above the CoW in healthy volunteers, with preliminary application in two Moyamoya patients.

**Results:**

In simulation, the IOES improved SNR efficiency by approximately 10% and used longer wavelength encodings that are less sensitive to subject motion. The effective labeling thickness was reduced using optimized PCASL parameters, which maintained high labeling efficiency. In healthy volunteers, these improvements allowed for the separation of at least nine arteries and their downstream tissues, with more accurate vessel decoding and closer alignment between the measured VEASL signal modulation and the encoding design. Vascular territories consistent with angiography were found in the Moyamoya patients.

**Conclusions:**

Combining IOES with optimized PCASL parameters, the vessel‐decoding efficacy in a region with complex vascular geometry above the CoW was improved. The automated encoding design process and scan times under 6 min make it feasible to observe flow patterns above the CoW in clinical settings, particularly for studies of collateral circulation.

## 1 INTRODUCTION

The ability to distinguish arterial blood supply to specific brain regions is crucial in the study of collateral blood flow in arterial stenotic diseases^1^ and the blood supply to lesions such as tumors or arteriovenous malformations.^2^ Vessel‐selective arterial spin labeling (ASL) can potentially achieve this noninvasively and without contrast agent, unlike conventional X‐ray–based approaches.^3^

Superselective and vessel‐encoded arterial spin labeling (VEASL) represent the two principal subcategories of contemporary vessel‐selective ASL techniques.^4^ Superselective ASL achieves good labeling efficiency by carefully positioning the labeling plane perpendicular to the targeted artery, allowing for the labeling of a single artery at a time, which simplifies both postprocessing and vessel planning.^5^ However, its SNR efficiency is significantly lower compared to VEASL when multiple target arteries are of interest.^6^ Therefore, the superselective methods are primarily confined to situations where only a small number of arterial branches are of relevance.^7^, ^8^, ^9^ VEASL instead modulates the signal from all blood vessels passing through the labeling plane to generate signals with specific encoding combinations.^10^, ^11^ Owing to its comparatively superior SNR efficiency, the requisite number of scan repetitions can be reduced, thereby enabling the data acquisition from multiple vessels, such as 13 arteries above the circle of Willis (CoW), to be accomplished within a clinically feasible scan time.^6^

However, an appropriate encoding scheme is required for VEASL to distinguish the blood arising from each of the feeding arteries. Due to the relatively complex positioning of arteries above the CoW, manual design of labeling schemes in this region is time‐consuming and will likely result in a suboptimal encoding.^6^ Random encoding has been proposed before,^12^ which does not need any precise planning, but the SNR efficiency is lower than planned approaches^13^ and does require the use of many encoding cycles, which may be infeasible for methods with longer scan times per volume, such as angiography. An alternative, the optimal encoding scheme (OES) automated approach,^13^ has been suggested, but this implicitly assumes that the vessels can be treated as single points within a 2D labeling plane. The high tortuosity of arteries above the CoW makes it challenging to meet this assumption by ensuring that all arterial branches are approximately perpendicular to the labeling plane throughout its thickness. Violating this assumption can result in reduced labeling efficiency and errors in vessel decoding.

In this work, to enhance the stability of VEASL encodings above the CoW, we propose improvements to the original OES method, optimizing its SNR efficiency while minimizing excessively high spatial frequencies, thereby reducing sensitivity to subject motion. Additionally, we optimized the pseudo‐continuous ASL (PCASL) parameters to minimize the effective thickness of the labeling plane (<6 mm) and ensure high labeling efficiency to overcome issues related to vascular tortuosity. We assessed the efficacy of vascular territory separation above the CoW by comparing our newly “optimized PCASL” parameters, combined with the improved OES (IOES) approach, to the conventional default parameters in combination with the original OES method.

## 2 METHODS

Simulations, phantom scans, and in vivo scans were all used to evaluate the effectiveness of IOES, combined with optimized PCASL parameters, for the application of labeling arterial branches above the CoW. All scans in this study took place on a Siemens 3 T Prisma (Siemens Healthineers, Erlangen, Germany) under a technical development protocol agreed by local ethics and institutional committees. A 32‐channel head RF receive coil was used in combination with the body coil for RF transmission.

### 2.1 IOES

In the original OES approach, the spatial location of the target vessels within the labeling plane is ascertained, and the corresponding number of columns is selected from a Hadamard matrix to form an ideal vessel‐encoding matrix. The OES method can then fully automate the identification of SNR‐efficient encodings using a Fourier‐transform approach to match the ideal encodings as closely as possible, regardless of the number or geometry of the vessels within the labeling plane.^13^

However, the OES method does not account for which columns to select from the Hadamard matrix or the ordering of those columns to optimize the SNR efficiency for the current vessel positions under all encoding combinations. In the case of four major arteries in the neck, we can manually determine how the ideal encoding matrix should be designed.^13^ Yet, for multiple arterial branches above the CoW, where the positional variations are quite complex between subjects, the ideal encoding combinations are difficult to design manually. In other words, it is impractical to definitively determine, for each encoding cycle, which arteries ought to be designated for labeling or control conditions to maximize the SNR efficiency across all potential encoding combinations for the given vascular geometry.

Therefore, in our proposed IOES method, assuming there are N vessels that need to be encoded, the first step is to randomize the ordering of the columns in the Hadamard matrix, selecting the first N columns (plus a column of ones to represent static tissue) as a provisional ideal encoding matrix. This is followed by the OES algorithm to derive the optimal encodings for the current provisional encoding matrix. Subsequently, by incorporating the actual PCASL parameters to be used in the acquisition, the in‐plane modulation of the longitudinal magnetization at each vessel position can be interpolated from a previously simulated spatial modulation function (see below).

Replacing the provisional ideal encoding matrix with this simulated encoding matrix allows for an assessment of the efficiency of the real encodings that could be achieved in practice. The condition number of this simulated encoding matrix, C, and the shortest spatial wavelength (λ) under this encoding design are calculated and plugged into Equation (1), which encourages low condition numbers and long wavelength encodings, which are more robust to subject motion. To allow adjustment for different cohorts of subjects with different motion properties, we define a maximum expected vessel displacement, M, set to 4 mm in this study.

(1) cost=1−1C22+1minλ/2M−122.

Note that the OES algorithm applies low‐frequency weighting in Fourier space, ensuring that half the spatial wavelength is always greater than M. Because the postprocessing in VEASL essentially involves the (pseudo)inversion of the encoding matrix, the condition number of the encoding matrix serves as a measure of the sensitivity of the solution to noise. For any actual encoding matrix, the condition number is always greater than 1. Thus, the closer the encoding matrix is to a Hadamard matrix, the closer the first term of the cost function is to zero. Note that the OES algorithm applies low‐frequency weighting in Fourier space, ensuring that half the spatial wavelength is always greater than M. In addition, as the minimum encoding wavelength approaches the expected motion during the scan, it will inevitably lead to imperfect vessel decoding. The second term of the cost function is designed to push the spatial wavelength much larger than M; otherwise, as it approaches M, the cost function becomes very large. This cost function therefore simultaneously optimizes for high SNR efficiency and the utilization of the longest possible encoding wavelengths, with its specific form chosen empirically as a smoothly varying function that strongly penalizes high condition numbers and wavelengths close to 2 M.

The IOES process iterates 100 times (each time with a different randomized ideal encoding matrix) to obtain the final encoding design with the minimum cost function. Note that the choice of Hadamard matrix used here always results in one encoding cycle being a nonselective control, but there is no nonselective label cycle. Whereas this would allow efficient signal decoding, for the purposes of assessing the IOES efficiency a nonselective label cycle was added during in vivo scanning. This allowed visualizing the nonselective perfusion signal and enabled the relative inversion efficiency to be calculated for the vessel‐encoded cycles. The IOES process was performed in MATLAB R2020a (MathWorks, Natick, MA). The calculation time for IOES with 100 iterations using a standard laptop was short: <50 s for nine vessels, which fitted easily into the time taken for other preliminary scans.

### 2.2 Bloch equation simulations

Both the OES and IOES methods implicitly require that the target vessels be treated as single points, which is challenging to uphold in the scenario of labeling above the CoW. The labeling process happens across a certain thickness,^14^ within which the vascular branches above the CoW are prone to extensive tortuousity,^15^ which may include segments aligned close to parallel to the labeling plane.^6^ Here, we use Bloch simulations to find a set of PCASL parameters that maintain high inversion efficiency while minimizing the effective thickness of the labelling plane to make the “point‐like” assumption more valid.

Conventional parameters for PCASL labeling were set similar to previous work: gradient amplitude during RF pulses (G_max_) of 6.0 mT/m, mean gradient (G_mean_) of 0.8 mT/m, RF duration of 500 μs, interval between the two RF pulses of 1000 μs, and flip angle of 20°, which were referred to as the *default settings*.^11^

To achieve thin‐slice labeling, the use of a long RF duration and/or high G_max_ is required, although care is needed to remain within hardware limitations and minimize peripheral nerve stimulation. To avoid RF amplifier fatigue at higher duty cycles, we fixed the RF interval at 1560 μs to allow longer RF durations while avoiding acoustic resonances on our scanner and off‐resonance sensitivity, trying to stick to around a 50% duty cycle. Subsequently, G_max_ was varied from 1.0 mT/m to 9.0 mT/m, and G_mean_ was varied from 0.1 to 0.9 mT/m. RF duration ranged from 50 μs to 950 μs, and the flip angle of the labeling pulses ranged from 8° to 30°. Default values for T_1_ of blood were set at 1650 ms, and T_2_ was 200 ms.^16^ The entire PCASL inversion process was investigated by Bloch equation simulations.^17^ A single spin was simulated traveling through the labeling plane; its final longitudinal magnetization was recorded; and the inversion efficiency was then calculated, correcting for T_1_ recovery. Finally, a parameter set was chosen to ensure that the thickness of the labeling plane, denoted by its approximate effective width as defined in Equation (3),^18^ was less than 6 mm and also avoided aliasing of labeling planes.

(2) z=2γ2π·Gmax·RF_duration<6mm.

It was also enforced that, after T_1_ recovery correction, the inversion efficiency was above 85% for laminar average flow velocities^19^ ranging from 5 to 50 cm/s. We prioritized the combination with the shortest RF duration to achieve minimum duty cycle, and then the parameters with the highest G_mean_ were selected from this subset, to avoid excessive pressure on the hardware due to large refocusing gradient amplitude, as our final optimized PCASL parameters, which was referred to as the *optimal settings*.

The effective flip angle across the labeling plane for a single RF pulse was simulated to clearly observe the difference between the default and optimal settings. The effect of vessel angulation through the labeling plane on the longitudinal magnetization Mz variation was also simulated using these two different PCASL settings assuming a spin velocity of 30 cm/s. For vessel‐selective labeling in VEASL, the inversion efficiency is spatially modulated across the labeling plane due to the additional transverse gradient blips,^10^ which could be applied using either an alternating positive and negative approach,^11^ termed *bipolar*, or a nonalternating way,^20^ known as *unipolar*. The spatial modulation, which we refer to as the inversion profile here, exhibits significant differences when using bipolar and unipolar approaches and also demonstrates entirely distinct responses to off‐resonance effects. Therefore, the impacts of off‐resonance on the inversion profile under unipolar and bipolar approaches were also simulated with selected optimized parameters.

### 2.3 SNR efficiency comparison

The theoretical SNR efficiency^10^, ^13^ for each territory was calculated based on simulating the encoding schemes generated by both the OES and the IOES for a blood velocity of 30 cm/s. The vessel coordinates from in vivo scans of seven subjects (see below) were used to compare the differences in theoretical mean SNR efficiency across three scenarios: OES with default settings, IOES with default settings, and IOES with optimal settings. The mean SNR efficiency was averaged from all territories. Paired t‐test was used to examine whether the mean SNR efficiency was improved.

To evaluate the robustness of IOES to motion compared to OES, we averaged the theoretical mean SNR efficiency variations across seven subjects as head motion increased in the three scenarios mentioned above. The motion translations and rotations were randomly perturbed, with translations sampled from a normal distribution and a SD ranging from 0 to 6 mm.

Additional simulations were conducted with higher duty cycles, reducing the RF interval from 1560 to 1380 μs and 1200 μs, corresponding to duty cycles of 55.8%, 63.0%, and 72.5%, respectively, while keeping other parameters fixed at the optimal settings. This was aimed at assessing potential performance improvements while possibly mitigating sensitivity to off‐resonance effects using shorter RF intervals. In keeping with in vivo B0 variation patterns, we assumed that only the anterior cerebral arteries (ACAs) experienced off‐resonance at 0, 50, and 100 Hz, whereas other vessels had no off‐resonance and assessed the mean simulated SNR efficiency differences across the three RF intervals. The spatial modulation of inversion efficiency for three RF intervals with optimal settings under three different off‐resonance conditions were first simulated. The SNR efficiency simulation was conducted for the IOES with optimal settings, using three different RF intervals. Paired t‐test was conducted to assess whether higher duty cycles led to a significant improvement in mean SNR efficiency.

### 2.4 Phantom scans

In order to experimentally confirm the labeling plane thickness under different PCASL parameters, a single‐slice scan with 2D single‐shot EPI readout was conducted, with the labeling plane positioned perpendicular to the imaging region (labeling duration = 600 ms, post‐labeling delay = 25 ms). Under the labeling condition, the region affected by the PCASL pulses induced a saturation effect,^13^ resulting in the appearance of a dark band.

### 2.5 In vivo healthy volunteer study

The spatial modulation of inversion efficiency for the default and optimal settings were measured in two volunteers (two male, age range 23–25 years), following the strategy of previous work (voxel size = 4.0 × 4.0 × 5.0 mm^3^, 20 slices, labeling duration = 1400 ms, post‐labeling delay = 750 ms, averages = 5, acquisition time ˜ 9 min).^20^ The center of the labeling was initially placed at the right internal carotid artery, whereas the center for the control was at the left internal carotid artery. Then, with a step size of 3 mm, the entire encoding pattern was shifted toward the right hemisphere, completing one full cycle. A pair of nonselective labeling and control images was also acquired within the same scan to enable the relative inversion efficiency in the right internal carotid artery and left internal carotid artery perfusion territories to be calculated for each shifted encoding pattern. The regions of interest for territories were manually delineated, avoiding the border zone region.

**FIGURE 1:**
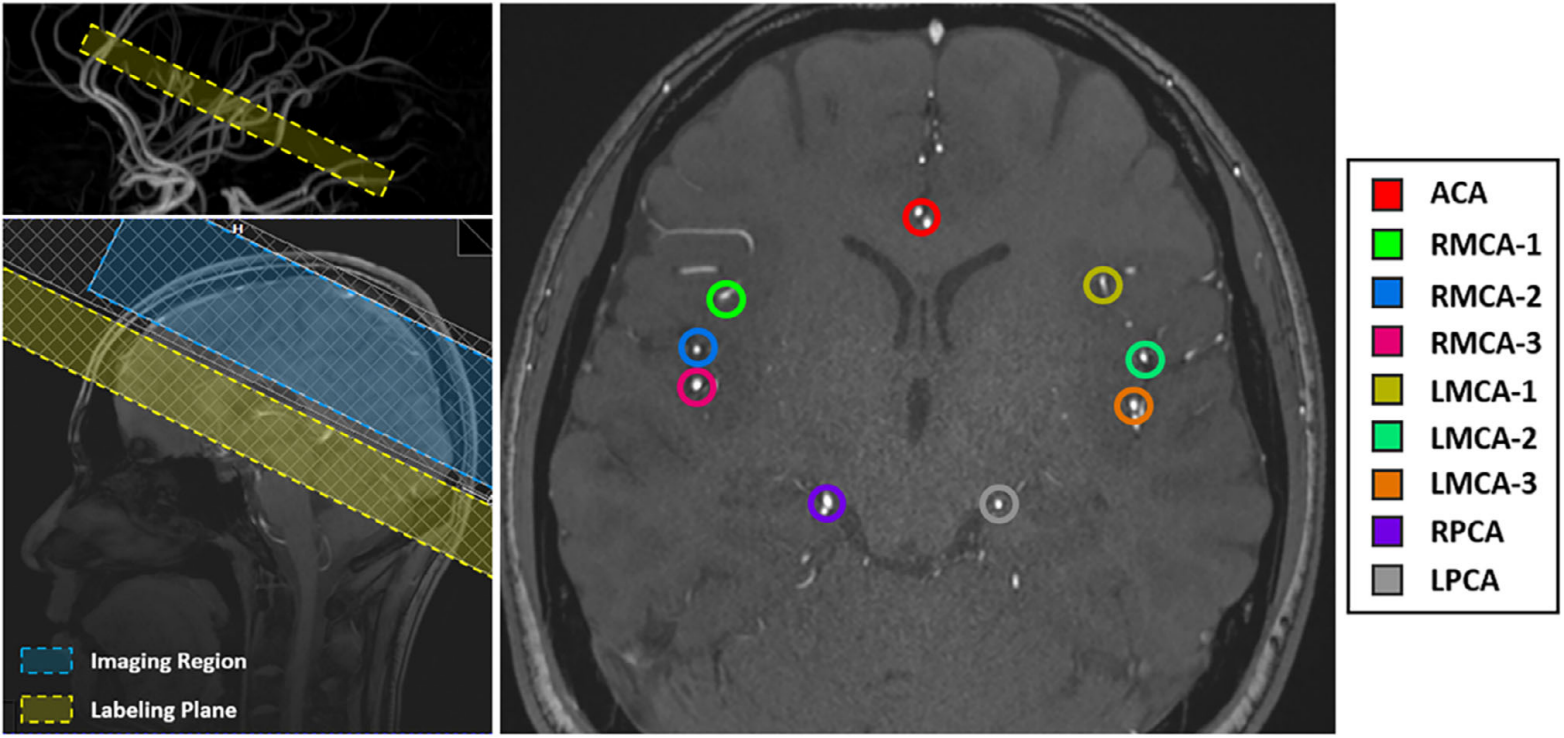
VEASL planning. TOF data from a representative volunteer showing the chosen labeling plane location just above the circle of Willis (yellow) overlaid on the sagittal MIP. During the scanning process, the displayed thickness of the labeling region was set to 30 mm, ensuring that there was a gap of at least 15 mm between the imaging region (blue) and the center of the labeling plane. The nine vessels to be encoded are highlighted with different colors. MIP, maximum intensity projection; TOF, time‐of‐flight; VEASL, vessel‐encoded arterial spin labeling.

To demonstrate the potential improvement of vessel encoding with the optimal settings and IOES, the following in vivo protocol was used. For planning, a 3D time‐of‐flight (TOF) image centered at the CoW was performed (voxel size = 0.6 × 0.6 × 0.6 mm^3^, 120 slices, TR = 21 ms, TE = 3.43 ms, acquisition time 4 min 10 s). The TOF images were loaded into the scanner console 3D viewer for selecting the labeling plane (Figure 1). The plane was rotated to be perpendicular to the A4 segment of the ACAs, distant from the frontal sinus, for initial coarse positioning. In the oblique transverse view, adjustments ensured three branches of the middle cerebral arteries (MCAs) and both posterior cerebral arteries (PCAs) were included, maximizing the angle between the plane and vessels. The ACAs were treated as a single artery if they were extremely close together, and as two separate arteries if the distance between them was several times the diameter of the ACAs. The coordinates of the feeding arteries were manually identified and their coordinates recorded for use within the OES and IOES calculation. Thirteen encodings were used, including 11 vessel‐selective encodings and nonselective label/control cycles. An additional MPRAGE^21^ structural scan (voxel size = 0.9 × 0.9 × 1.0 mm^3^, TR = 2000 ms, TE = 2.11 ms, TI = 880 ms, acquisition time 4 min 54 s) was also acquired for tissue segmentation and image analysis.

Seven volunteers (six males, one female; age range 23–26 years) were scanned with VEASL 2D multi‐slice EPI vascular territory imaging (VTI) (acquisition time 5 min 59 s), and three of these were also scanned with the dynamic thick‐slab 2D projection balanced steady‐state free precession angiography^22^ (acquisition time 2 min), with one scan using the default settings combined with OES and the other using the optimal settings combined with IOES. An additional four healthy volunteers (one male, three females; age range 22–32 years) were scanned with both VTI and dynamic 2D angiography, including a combined scenario using IOES with default settings, which allowed us to evaluate: a) the improvement of IOES over OES with both using the default settings, and b) the improvement of IOES with optimal settings over the default settings. An additional scan was performed in these four subjects, with the RF interval shortened to 1380 μs, to evaluate whether vessel‐decoding performance would improve by increasing the duty cycle to 63%. All scans were performed in a randomly assigned order. The background suppression and readout parameters were similar to previous publications and are listed in Table S1. The bipolar VEASL approach was chosen for the in vivo scans, based on simulation results (see below). The imaging regions were positioned parallel to the labeling plane, with a gap more than 15 mm, to reduce static tissue saturation effects for the bottom slices.^6^ The B_0_ shimming region covered both the labeling plane and imaging regions. One of the subjects underwent an additional scan to assess the impact of shimming only for the imaging region, particularly on the blood signal arising from the ACAs, where B_0_ homogeneity might tend to be poorer.

### 2.6 Moyamoya patients

To demonstrate the feasibility of this approach in a clinical cohort, two patients with Moyamoya disease (one male, one female; mean age, 51 years) at different stages were scanned with IOES using optimal setting and TOF angiography. The X‐ray digital subtraction angiograms were also collected as part of the standard clinical pathway. Patient informed consent and institutional ethical and committee approval were obtained in advance for MRI scans. One preoperative patient with significant left middle cerebral artery stenosis and occlusion, and another postoperative patient who underwent right‐sided bypass surgery with superficial temporal artery‐to‐middle cerebral artery (STA‐MCA) anastomosis combined with dural patching. Due to significant variations in some MCA arterial branches, the encodings used were modified in each case to those that were observable. The patients were only scanned with the VTI protocol, using parameters almost identical to those of the healthy volunteer protocols. The variability in the number of identifiable vessels, which might arise from the disappearance of anterior circulation arteries or the formation of collateral pathways, led to the use of a different number of cycles. For more details, please refer to Figures 8 and 9.

### 2.7 Image analysis

The vessel‐decoding process was performed using a *maximum* a posteriori solution^23^ within a Bayesian framework.^24^ As mentioned above, the analysis included nine arterial branches. Two vessels per class were used, allowing probabilistically for mixed blood supply, although this was not expected to be commonly observed in healthy individuals above the CoW. The extracranial arteries were prominently visible in the angiography dataset, such as the left and right superficial temporal arteries (STAs) and occipital arteries. After considering these additional four vessels, we conducted a second analysis of the angiography dataset in healthy volunteers, including these vessels in the decoding process, even though they were not explicitly included in the encoding design. In postoperative Moyamoya patients, the STA on the surgical side was incorporated into the decoding process to assess the perfusion territory of this specific bypass artery. We also reprocessed VTI data from two healthy volunteers and one preoperative Moyamoya patient using fewer averages, which led to shorter VEASL acquisition time, to evaluate whether vessel decoding performance still maintained good quality.

Nonselective images in VTI were used to compare the relative labeling efficiency under different PCASL parameter combinations, using SNR as a proxy for labeling efficiency. For a fair comparison, structural images were segmented to extract the gray matter (GM) region and then registered to the VTI space. A GM mask was obtained using a threshold of 0.7. The SNR of the nonselective images was then calculated by dividing the mean signal within the GM mask by the SD within a manually delineated background mask.

To assess whether signal variations in a specific territory followed the expected encoding pattern, we extracted the mean signal within each encoding cycle from manually drawn masks for specific vascular territories. This mask was then intersected with the GM mask obtained previously to ensure that the signal was primarily coming from GM. For the angiography dataset, the high SNR in arteries facilitated the extraction of a vascular mask by applying a straightforward empirically chosen threshold. The mean relative inversion efficiency within the mask for each vessel‐encoded image was then calculated by comparison to the nonselective PCASL data as follows:

(3) $$\begin{array}{ll} & \text{relative inversion efficiency}=\\ & \;\frac{\text{nonselective control}-\text{selective label}}{\text{nonselective control}-\text{nonselective label}}. \end{array}$$

## 3 RESULTS

### 3.1 IOES

**FIGURE 2:**
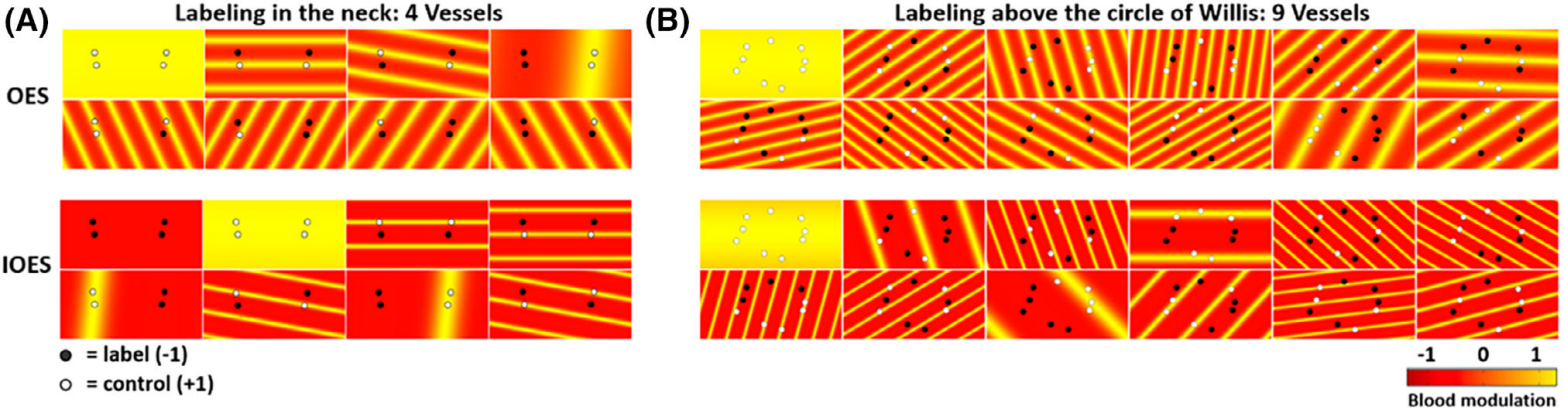
Optimal encoding scheme simulations, showing the vessels targeted to be in the label (black) or control (white) conditions and corresponding encodings calculated by both the original OES and proposed IOES approaches. (A) Four vessel encodings: IOES achieved a near‐perfect Hadamard encoding using much lower spatial frequency encodings, with wavelengths ranging from 18.29 to 101.89 mm, that are more robust to motion, whereas the original OES method chose suboptimal encodings, with wavelengths ranging from 16.45 to 101.89 mm. The average wavelength of IOES increased from 26.90 mm in OES to 51.14 mm. (B) Nine vessel encodings above the CoW: The IOES clearly reduced the average spatial frequency of the encodings, with wavelengths ranging from 20.23 to 120.68 mm, compared to 17.24 mm to 49.73 mm for OES. The average wavelength increased from 26.58 mm in OES to 38.97 mm in IOES. CoW, circle of Willis; IOES, improved optimized encoding scheme; OES, optimized encoding scheme.

As shown in Figure 2A, in the scenario of labeling the four major brain‐feeding arteries in the neck, the distribution of vessels approximates a rectangle pattern. IOES achieved a near‐perfect Hadamard encoding using much lower spatial frequency encodings that were more robust to motion, whereas the original OES method chose suboptimal encodings. Figure 2B presents the vessel distribution above the CoW from one in vivo scan. The IOES effectively lowered the average spatial frequency of the encodings, making it more robust to head motion. However, there were still some vessels in the transition zone between the label and control regions, and partial labeling was difficult to avoid completely due to the complex vessel geometry above the CoW.

### 3.2 Optimized PCASL parameters

**FIGURE 3:**
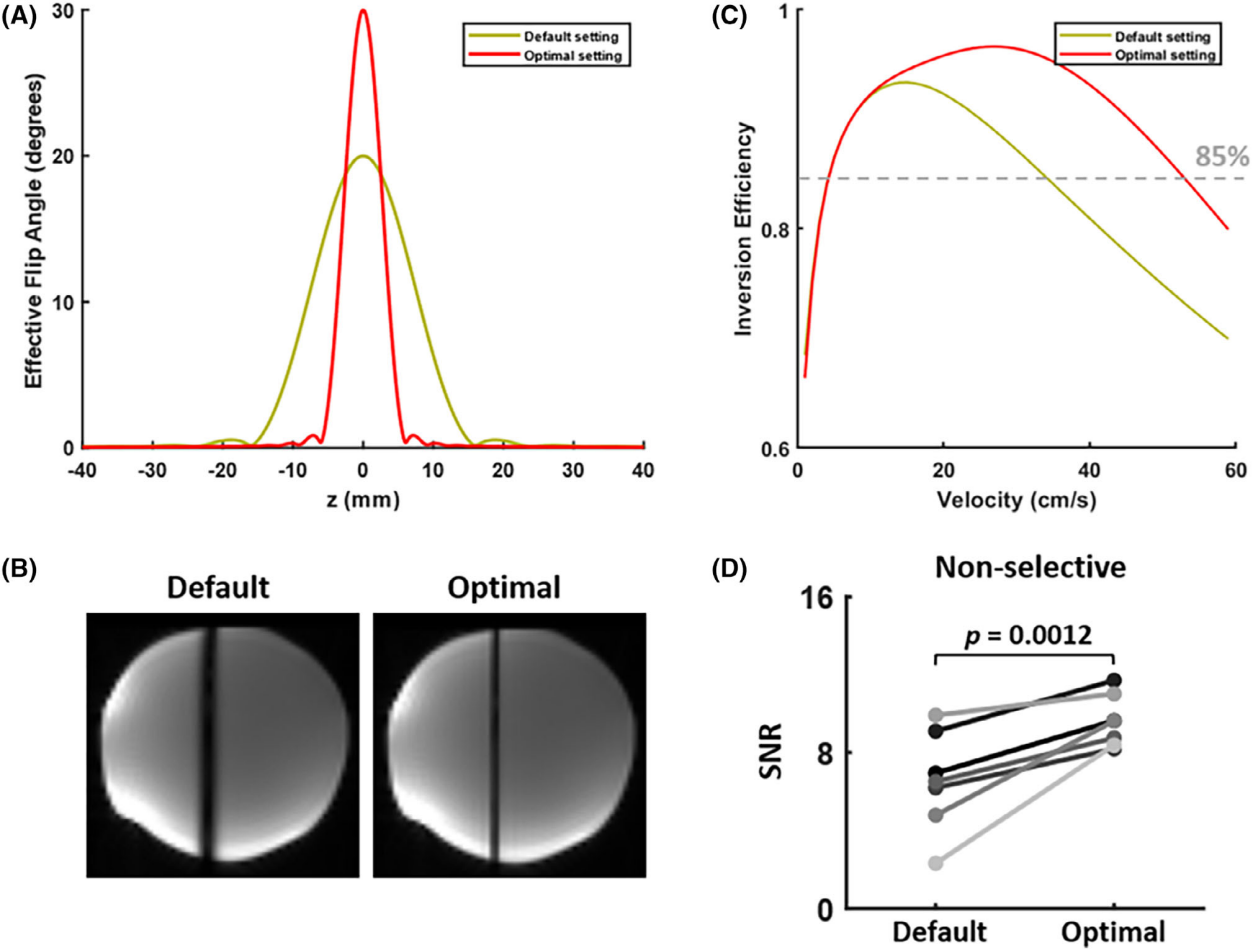
Optimization of PCASL parameters. (A) Simulated flip angle across the labeling plane for the default and optimal settings. (B) Phantom results, with saturated signal (dark vertical bands) giving an indication of the labeling plane thickness. (C) The simulated inversion efficiency was improved with optimal settings, which could maintain a high labeling efficiency over a broader range of blood flow velocities. (D) In the in vivo data, the SNR of the nonselective perfusion signal was improved with the optimal settings. Different lines represent different subjects. PCASL, pseudo‐continuous arterial spin labeling.

Based on the results from simulation studies, the optimal parameters were chosen as follows: G_max_ of 9 mT/m, G_mean_ of 0.45 mT/m, labeling RF pulse duration of 870 μs, interval of 1560 μs, and flip angle of 30°. The criteria we chose prioritized ensuring that the RF duration was as short as possible to reduce the duty cycle, with flip angle and G_max_ both at the simulated upper limits. Compared to the default settings, using the optimal parameters, the effective labeling thickness was thinner (Figure 3A,B) and could ensure higher inversion efficiency at higher blood flow velocities (Figure 3C). This was confirmed in the in vivo results, with a significant improvement in SNR within the GM observed in nonselective perfusion images (*p* = 0.0012, Figure 3D). Whether using the default or optimal PCASL settings, a relatively high labeling efficiency is maintained even at a 60° angle, as shown in Figure S1.

Unipolar VEASL leads to a very narrow label region based on the optimized parameters (Figure S2), which could make achieving an efficient encoding more difficult as well as more sensitive to subject motion. In the presence of field inhomogeneity, it can also lead to shifts in the encoding pattern (Figure S2). Given the relatively complex distribution of vessels above the CoW, and their potential proximity, a gradual transition between labeling and control was preferred. Therefore, only the bipolar approach was used for in vivo scanning. Simulations revealed a close similarity between the inversion profiles under default or optimal parameters using the bipolar approach, aligning with the in vivo results (Figure S3).

### 3.3 Theoretical SNR efficiency

**FIGURE 4:**
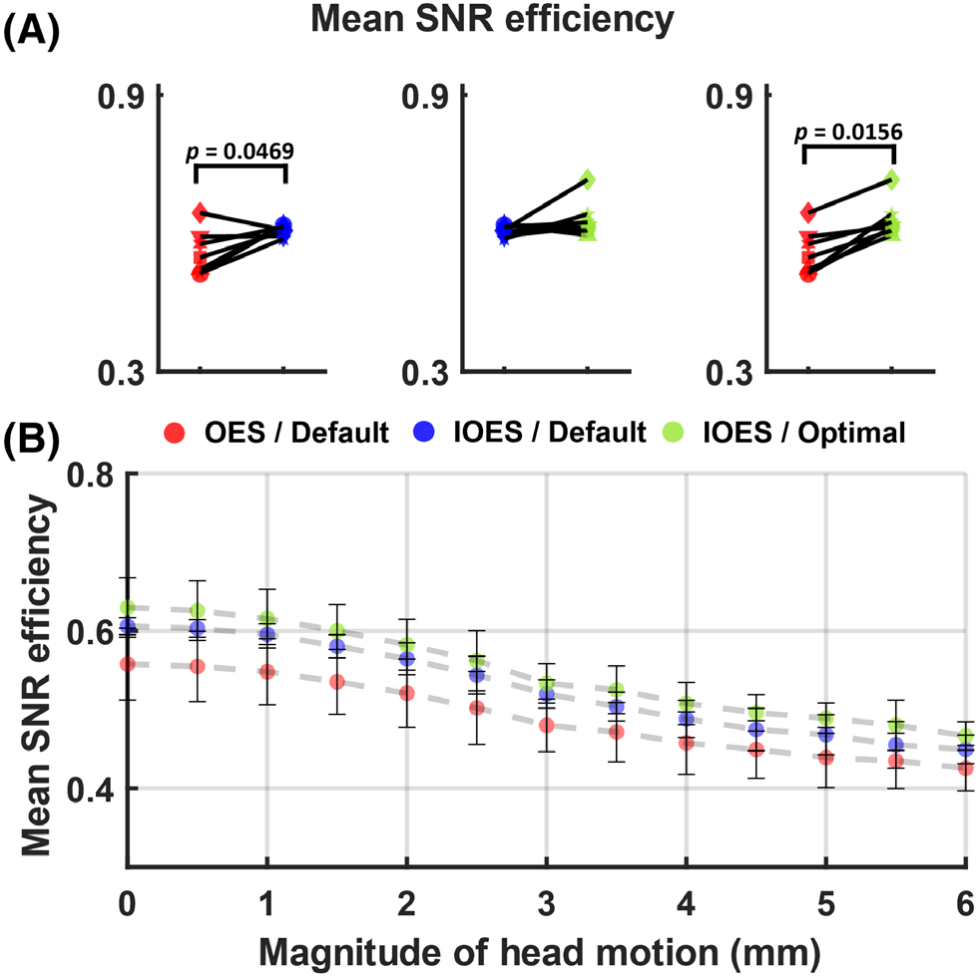
Theoretical SNR efficiency comparison for nine vessels above the CoW. (A) When using default parameters, IOES provided a significant improvement over OES. No significant difference was observed in simulation between the optimal and default settings when both used IOES. Combining IOES with optimal parameters resulted in a significant enhancement in SNR efficiency compared to OES with default settings. (B) The SNR efficiency of IOES, whether using the default or optimal setting, yielded higher performance in the presence of motion between the planning and VEASL acquisitions compared to OES with the default setting.

The theoretical SNR efficiency of OES and IOES was calculated for each territory, respectively, based on the simulated inversion efficiency for each vessel during each encoding cycle. As shown in Figure 4A, IOES showed a significant improvement over OES when using default parameters (*p* = 0.0469), with an average increase of 8.6%. With IOES, the optimal parameters yielded a 3.9% average improvement over the default parameters, although this was not statistically significant. The combination of IOES with optimal settings resulted in a substantial enhancement in SNR efficiency over OES with default settings (*p* = 0.0156), with a mean improvement of 12.9%, from 55.8% reaching up to 63.0%. Furthermore, regardless of whether default or optimal settings were used, IOES consistently outperformed OES with default settings in terms of SNR efficiency in the presence of motion between the planning scan and the VEASL scan (Figure 4B), suggesting that IOES was more robust to motion.

Increasing the RF duty cycle had a minimal impact on the shape of the spatial modulation, leading only to small increases in labeling efficiency at 50 or 100 Hz off‐resonance (Figure S4A). Without off‐resonance, the SNR efficiency was 0.630 ± 0.038 for the 1560 μs RF interval, 0.638 ± 0.069 for the 1380 μs RF interval, and 0.578 ± 0.038 for the 1200 μs RF interval. At 50 Hz off‐resonance, the corresponding SNR efficiency was 0.607 ± 0.039, 0.617 ± 0.068, and 0.563 ± 0.037, respectively. At 100 Hz off‐resonance, the corresponding SNR efficiency was 0.565 ± 0.040, 0.573 ± 0.064, and 0.525 ± 0.035, respectively. The paired t‐test demonstrated that increasing the duty cycle did not necessarily improve the final SNR efficiency at off‐resonances of 50 or 100 Hz, as shown in Figure S4C.

### 3.4 Vessel‐decoding performance

**FIGURE 5:**
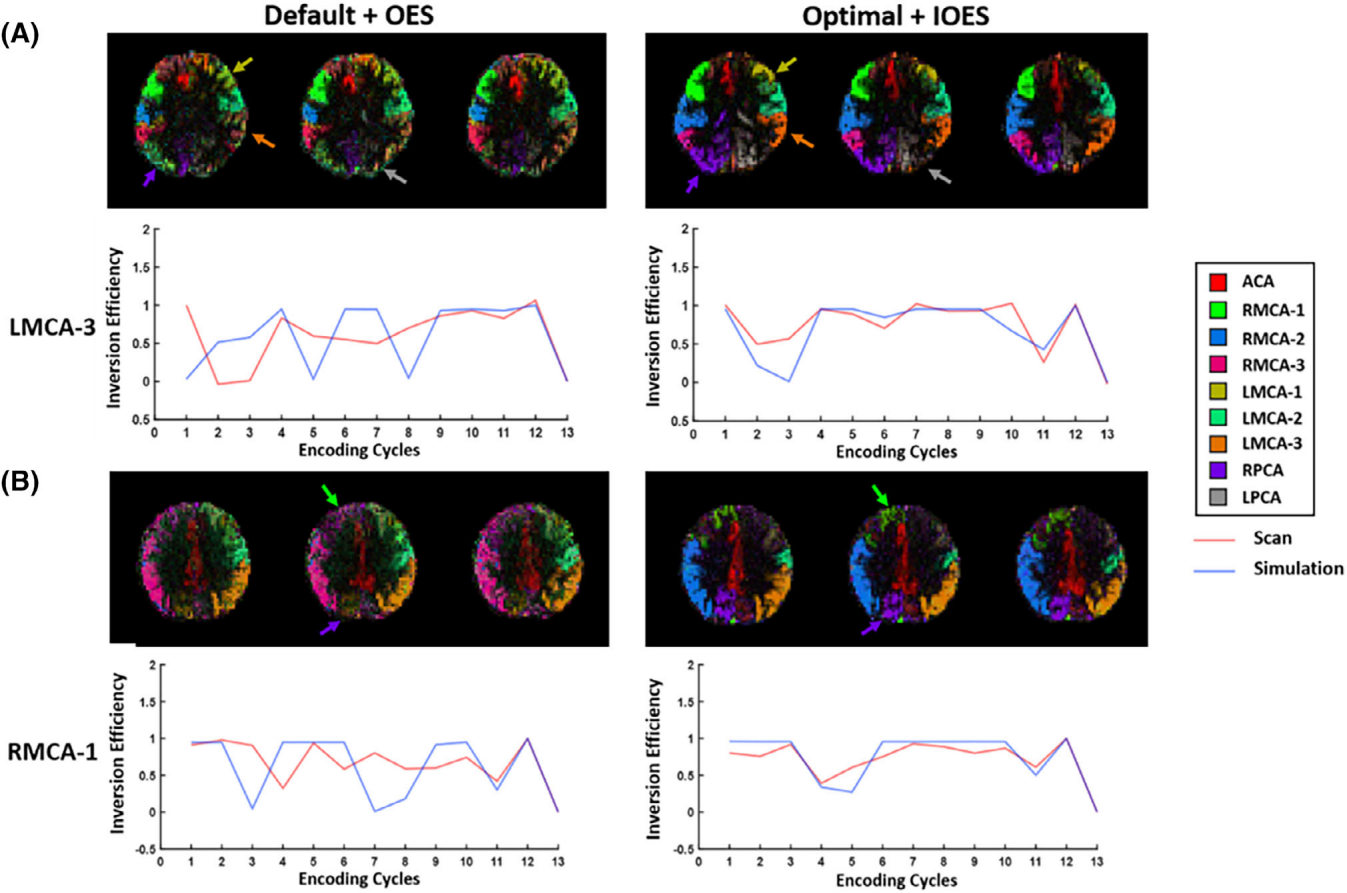
Example vascular territory maps above the CoW. (A) Using the optimal parameters and IOES, data from this subject showed a noticeably better delineation in the territories of the LMCA, RPCA, and LPCA, reducing instances of mixed blood supply. (B) Another subject also showed much cleaner separation of vascular territories, especially in the territory of RMCA‐1 and RPCA. The more distinctly separated territories, such as LMCA‐3 in the first subject and RMCA‐1 in the second subject, showed a superior alignment between actual and simulated signals when compared to the default and OES combination, likely due to reduced vessel tortuosity within the thinner labeling plane, enabling these territories to be more effectively separated. Perfusion signal and arrow colors correspond to different vascular territories (see legend).

**FIGURE 6:**
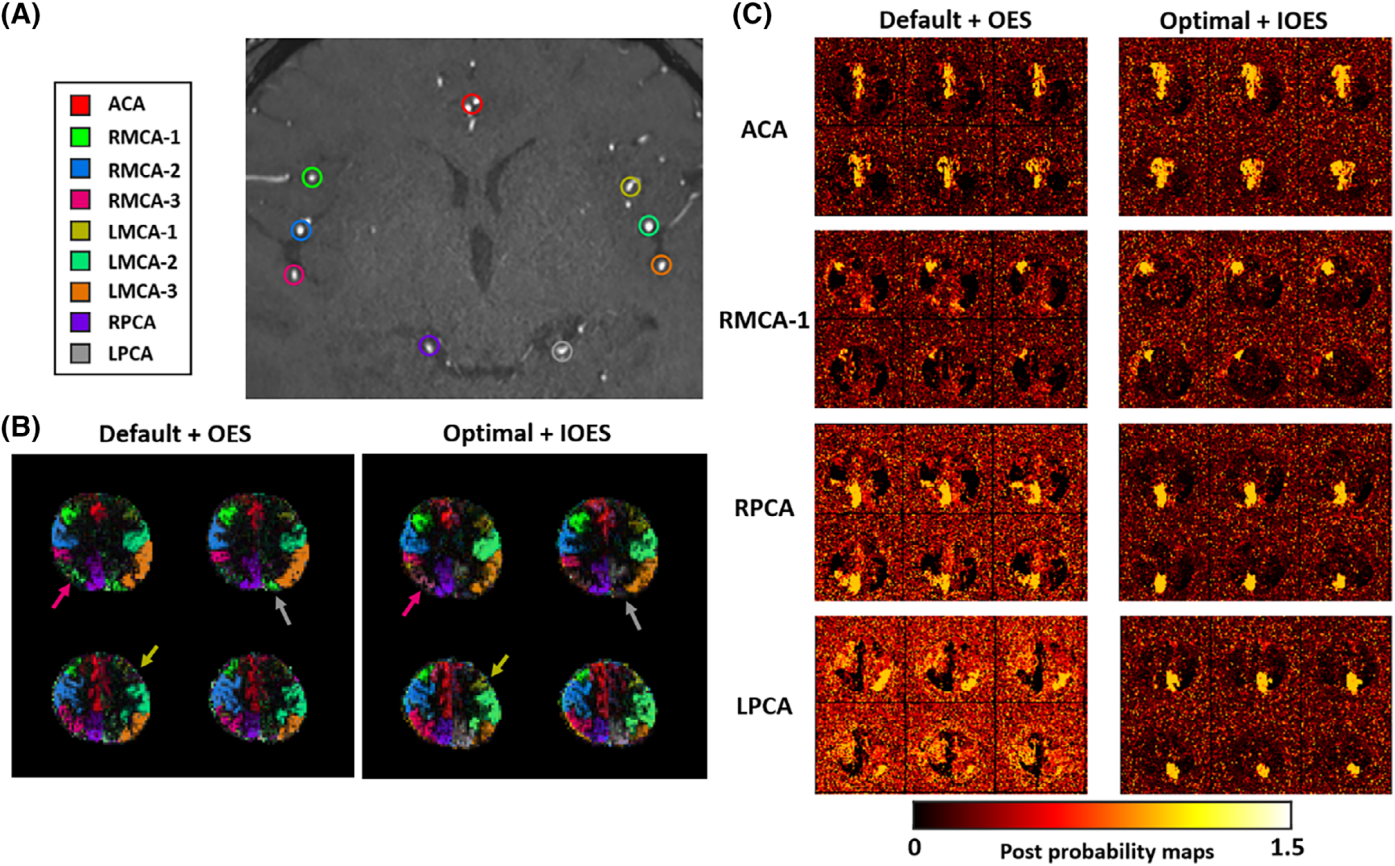
Example vascular territory and Bayesian probability maps another subject. (A) The nine vessels to be encoded within the labeling plane. (B) Using the default PCASL settings with OES, the territory of RMCA‐1 (green) appeared in the LPCA territory, which should be gray, indicating an obvious error. With the combination of optimal PCASL settings and the IOES, the vascular territory maps effectively mitigated misclassification issues between the right MCA segments and LPCA. (C) The Bayesian analysis probability maps demonstrated a more robust estimation of the vascular territories for these four example arterial branches, with higher probabilities in the expected anatomical locations. For example, the PCAs were less likely to show inappropriate partitioning, whereas the background probability was also reduced.

Example vascular territory maps and signal variations within selected territories are shown in Figure 5A,B. The optimal parameter combination with IOES enables more precise vessel decoding, resulting in clearer boundaries and fewer occurrences of mixed blood supply, which are not expected in these healthy volunteers. In Figure 5A, the LPCA territory achieved better separation. Both left MCA‐2 and LMCA‐3 had clearer demarcations, with inversion efficiency variations in LMCA‐3 aligning more closely with IOES design, contributing to accurate separation. This was likely due to reduced vessel tortuosity within the thinner labeling plane, improving the accuracy of treating each vessel as a single point in a 2D plane. In another subject (Figure 5B), although perfect vessel decoding of all territories was not achieved, the optimal parameters with IOES still outperformed the default parameters with OES. For instance, right MCA‐1 resembled simulated signals more closely. Similarly, Figure 6B shows favorable vessel decoding with the optimal parameters and IOES. Probability maps (from the Bayesian framework) showed clearer blood supply predictions with the optimal/IOES approach. For instance, RMCA‐1 had more concentrated probability in the expected anterior region, whereas the PCAs exhibited fewer incorrect partitions (Figure 6C).

**FIGURE 7:**
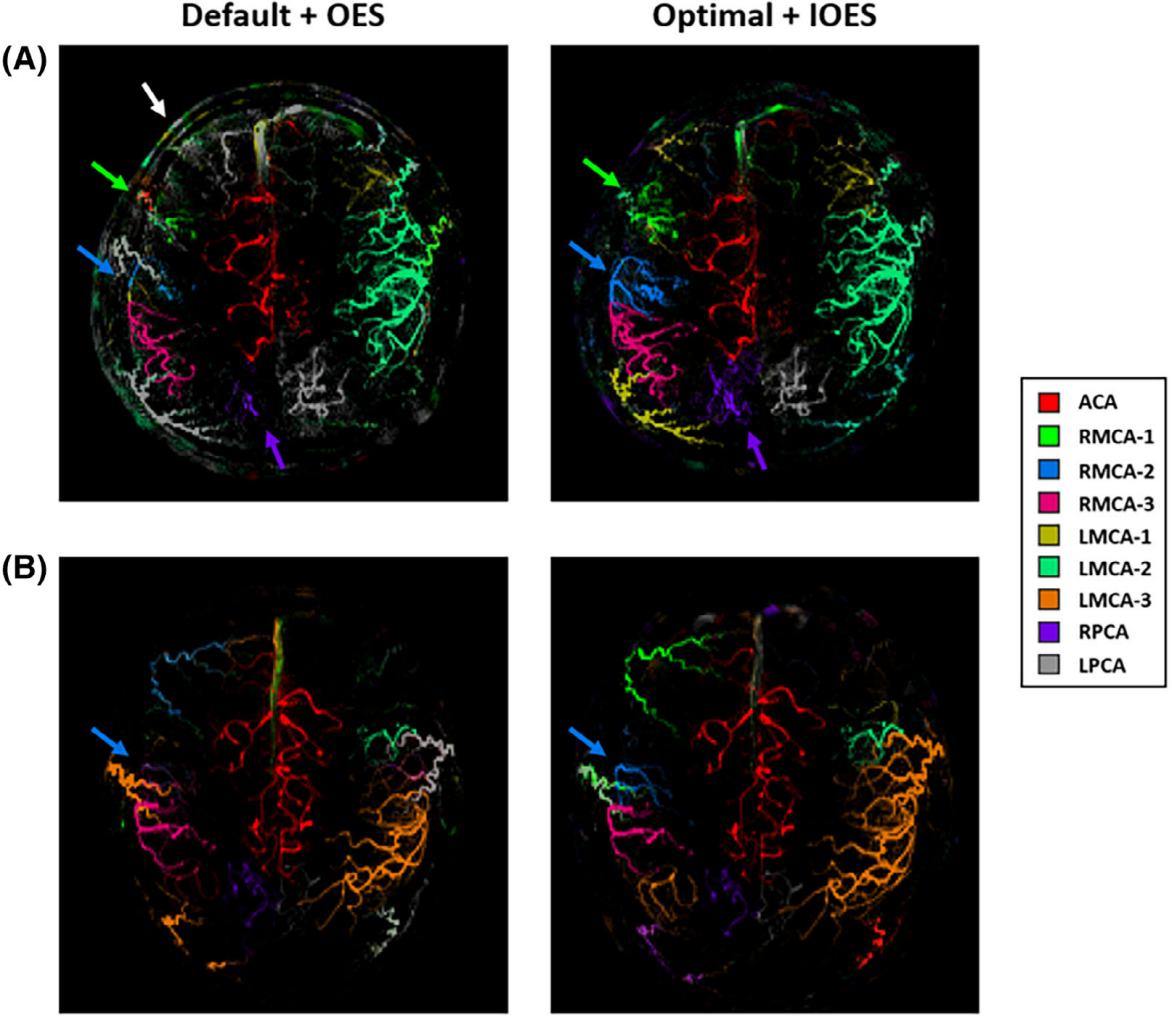
Example VEASL angiographic data in two subjects. (A) Due to the thicker labeling plane of the default parameters, its perturbation of the static tissue signal within the imaging region was more pronounced, and it did not perform well in imaging multiple arterial branches on the right side, which were better delineated with the optimal parameters and IOES approach. (B) In this subject, the vessel separation was comparable, except for a notable signal enhancement observed in the combination of optimal and IOES, particularly in RMCA‐2 (blue arrow). White arrows represent signals from tissues such as the scalp. Note that the extracranial vessels were not explicitly included in the vessel‐decoding process here, and therefore are assigned to whichever vessel had the most similar encoding.

Similar trends were also seen in the angiography data (Figure 7A,B). Moreover, the optimized parameters, by reducing the thickness of the labeling plane, effectively mitigated the impact of the labeling pulses on the imaging region, thus preventing the influence of tissue signal perturbations on the subtracted angiogram (Figure 7A). It was also possible to achieve more reliable separation of the ACAs when they were spatially distinct within the labeling plane (Figure S5). The benefit of the optimal settings over the default settings could be better demonstrated in the additional dataset, which included another combination of IOES with default settings, showing more plausible vascular territories due to the reduced sensitivity to tortuous vessel anatomy (Figure S6). When including extracranial arteries into the analysis, the combination of optimal parameters and IOES not only ensured accurate separation of the nine intracranial arteries but also had the potential to simultaneously separate a total of 13 vessels, including both intracranial and extracranial arteries (Figure S7). Even in the presence of considerable head motion during the scan, this combination could still separate several arterial branches (Figure S8). However, when the RF interval was shortened to 1380 μs and IOES was applied, vessel‐decoding performance degraded in each subject, with some cases resulting in decoding errors, whereas much better territory images were obtained using the original optimized parameters with a duty cycle close to 50% (Figure S9).

**FIGURE 8:**
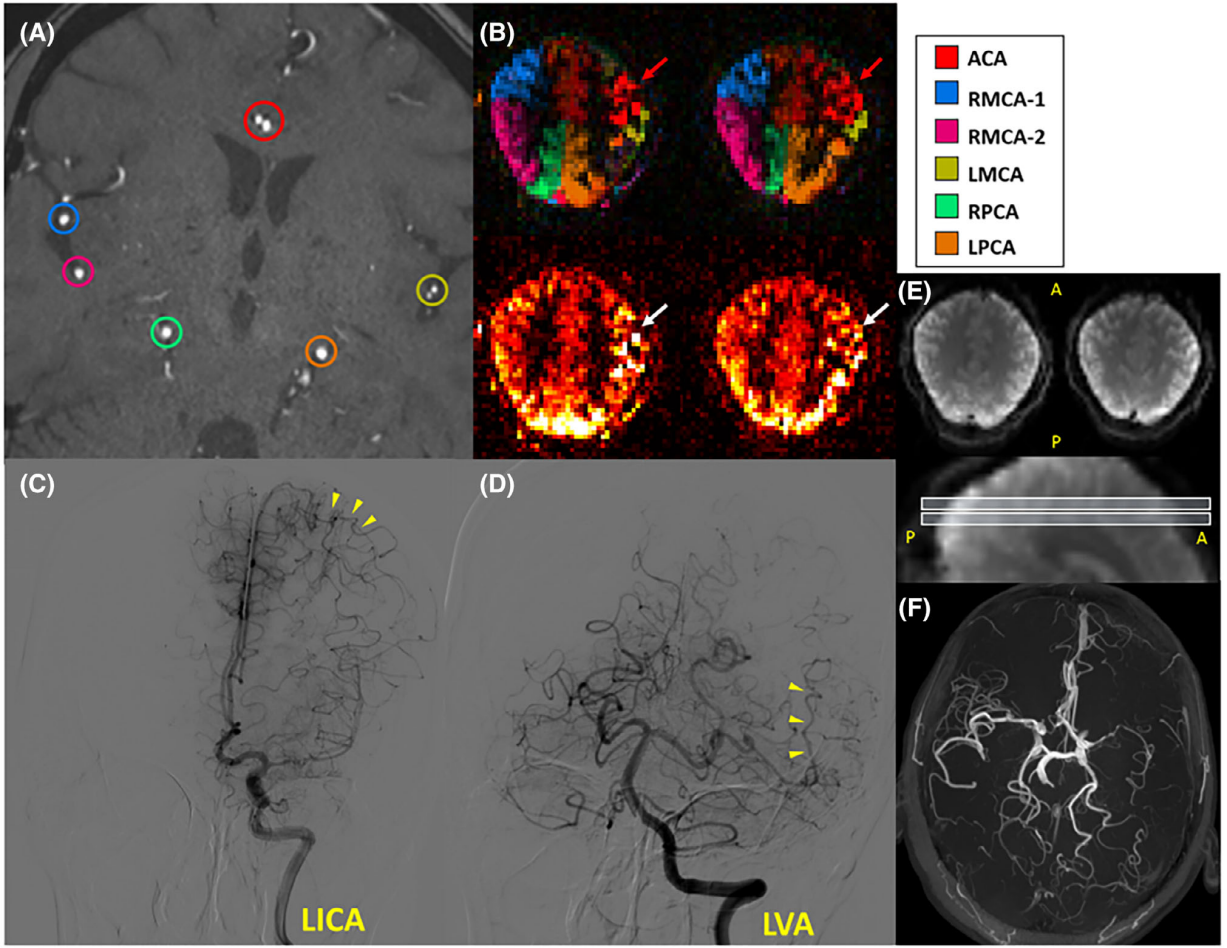
The vascular territories above the circle of Willis in the preoperative Moyamoya patient. The TOF image (F) showed severe LMCA stenosis and occlusion, with only one LMCA branch and two RMCA branches visible within the labeling plane (A), limiting the encoding process to six vessels. The encoding matrix was based on an 8D Hadamard matrix with a nonselective label, resulting in nine cycles. The anatomical images (E) demonstrated the positioning of the two displayed imaging planes. The nonselective perfusion‐weighted images (B, bottom row) showed prominent arterial transit artifacts (white arrows) in LMCA territories, indicating delayed blood arrival from potential collateral flow. It is clearly observed that the territories of the ACA and LPCA have extended into the MCA territory (B, top row), indicating the establishment of multiple collateral circulation pathways that correlate with DSA findings (yellow arrows), shown as coronal views after contrast injection into the LICA and LVA (C, D). ACA, anterior cerebral artery; DSA, digital subtraction angiogram; LICA, left internal carotid artery; LMCA, left middle cerebral artery; LPCA, left posterior cerebral artery; LVA, left vertebral artery; MCA, middle cerebral artery; RMCA, right middle cerebral artery.

**FIGURE 9:**
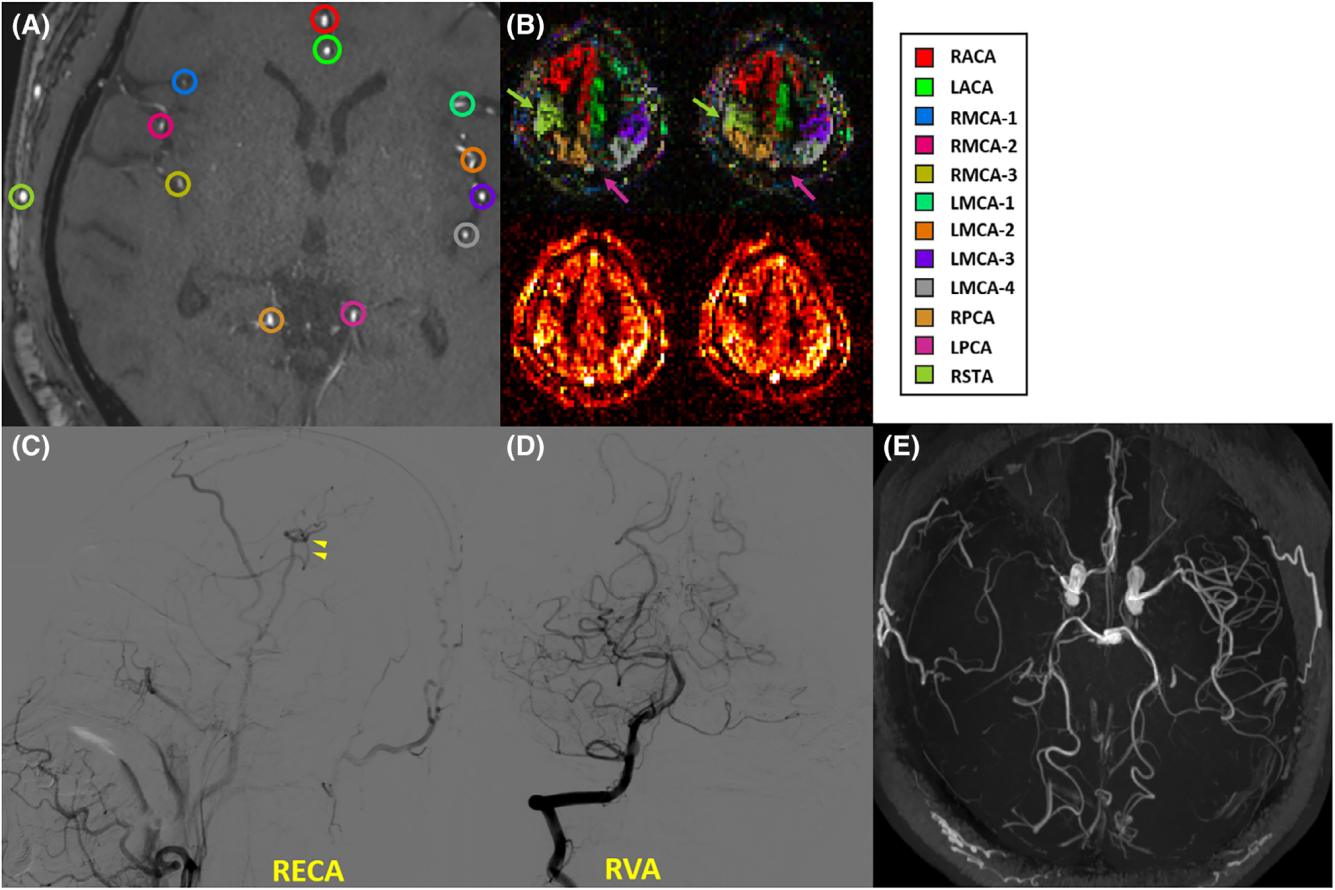
The vascular territories above the circle of Willis in the postoperative Moyamoya patient. This patient with severe RMCA stenosis (as shown in the TOF, E) underwent right‐sided bypass surgery with STA‐MCA anastomosis, combined with dura patching. Within the labeling plane (A), the RMCA branches were visible but with very weak signal intensity, whereas the LMCA branches appeared highly irregular, complicating the planning process. Ultimately, the intracranial arterial branches, a total of 10 vessels, were identified as targets, and the RSTA was only included in the vessel‐decoding process. The intracranial vascular territory of RSTA was clearly established after the surgery (B, green arrows), and the RPCA also showed a tendency to compensate for the territory of RMCA. The left and right ACAs were also well distinguished. However, LPCA decoding failed (pink arrows), likely due to the labeling plane's position, which, upon reviewing the TOF images, was nearly parallel to the LPCA, resulting in insufficient labeling. Although the DSA images (C, D) were of moderate quality, the data acquired after contrast injection into the RECA clearly demonstrated patent and hypertrophied bridging arteries, confirming the flow of blood from the RSTA into the brain, as seen with VEASL, whereas the RVA data did not show significant collateral flow toward the RMCA territory. The information presented was less intuitive compared to VEASL. RECA, right external carotid artery; RPCA, right posterior cerebral artery; RSTA, right superficial temporal artery; RVA, right vetebral artery; STA‐MCA, superficial temporal artery–middle cerebral artery.

Vascular territory maps of two Moyamoya patients were shown in Figures 8 and 9, demonstrating good correspondence between VEASL results, TOF angiograms, and clinical status. In the preoperative patient, the ACA and LPCA were observed to compensate for the territory of the highly stenosed LMCA (Figure 8B). In the postoperative patient, the territory of the right superficial temporal artery was clearly visible, indicating the success of the surgery (Figure 9B). Furthermore, when using fewer averages (scan times <4.5 min), both healthy volunteer and patient data showed similar decoding performance, albeit with a slightly lower SNR (Figure S10).

The optimized parameters utilized a longer RF interval, which should be more sensitive to field inhomogeneity. When the shimming volume did not include the labeling plane, the right ACA signal was reduced in the subject (Figure S11), confirming that shimming for both the labeling plane and imaging region is important when using these optimized parameters.

## 4 DISCUSSION

In this study, we have demonstrated that the PCASL parameters can be optimized to efficiently reduce the effective thickness of the labeling plane while increasing labeling efficiency. This thinner labeling plane reduces the impact of vessel tortuosity within the labeling region, improving the validity of treating each vessel as a single point when calculating the optimal vessel encodings, especially for complex vascular morphology above the CoW.

Combining the optimized parameters with the proposed IOES approach led to significant improvements in SNR efficiency and vessel‐decoding ability for vessel‐encoded perfusion and angiography data. This improvement can be attributed to: a) the reduced probability of vessels exhibiting intricate curvature within the thinner labeling region, aligning the actual ASL signal modulation within vessels or corresponding downstream tissues more closely with the IOES design; and b) the selection of lower spatial frequency encoding patterns that better fit to the vascular geometry of the subject, making this approach more SNR‐efficient and reducing motion sensitivity. The improved vessel‐decoding power from the combination of IOES and optimal parameters is also evident from the outputs of the Bayesian analysis, leading to more spatially specific probability maps. When including the extracranial arteries in the angiographic analysis, the IOES/optimal parameter combination seemed better able to separate these signals, even though these additional vessels were not explicitly considered during the encoding design. This was perhaps due to the longer spatial wavelengths used during the encoding process, giving a more distinct signal variation within these distant vessels. The optimal/IOES approach also seemed to be less affected by head motion, again perhaps due to the longer spatial wavelengths used for encodings, although further work is required to confirm these results robustly.

One key difference between IOES and the original OES method is that OES optimizes encoding for a single ideal matrix without considering practical ease for a given vascular geometry. IOES, on the other hand, examines many possible ideal matrices, selecting the most practical with long wavelength encoding patterns. Another key difference is that IOES explicitly incorporates a realistic spatial modulation function and the resulting SNR efficiency into its design process, producing more SNR‐efficient encodings.

Simulations suggested that using the bipolar VEASL approach would be more robust when using a relatively thin labeling plane because the label and control regions of the spatial modulation function (Figure S2) are relatively broad, making it easier to achieve the desired vessel encoding for complex vascular geometries. In addition, the presence of off‐resonance only reduces the labeling efficiency, and this can be mitigated by shimming for the labeling plane and imaging region simultaneously. The alternative unipolar approach results in a very narrow labeling region within the spatial modulation function, making it more difficult to position the vessels to be labeled within this narrow region for arbitrary vessel geometries—and also making it vulnerable to labeling errors with even minor head motion. In either of these scenarios, this would introduce a mismatch between the acquired and simulated signals and therefore potential misclassification of the ASL signal. Furthermore, the unipolar approach is sensitivity to field inhomogeneity that leads to a direct shift in the encoding pattern, whereas bipolar only reduces inversion efficiency without altering the position of labeling bands (Figure S2). Notably, the ACA signals, where off‐resonance effects are typically most problematic due to proximity to the frontal sinus, seem robust at 3 T when using the optimal settings with the shimming region incorporating the labeling plane, and the relative inversion efficiency aligns well with simulations (Figure S9). This is because we chose the convergence point of the left and right ACAs as the labeling point, treating ACAs as a single artery. This position, typically distant from the tissue–air interface, combined with the bipolar approach, thus ensuring that a robust encoding of these arteries is achieved.

Given the longer RF interval, the optimized parameters are more sensitive to off‐resonance. However, increasing the duty cycle by shortening the RF interval only resulted in a slight improvement in inversion efficiency (Figure S4). Higher duty cycles can also cause RF amplifier drift across scanners,^25^ negatively impacting labeling efficiency in the PCASL pulse train. Furthermore, as the duty cycle increases, spatial modulation deviates more from the cosine function. Because both OES and IOES rely on Fourier‐based methods, they assume spatial modulation should approximate a cosine function. Without this, the validity of OES/IOES is compromised, which likely explains why higher duty cycles did not improve SNR efficiency (Figure S4) and even reduced vessel‐decoding performance (Figure S9). In fact, when we positioned the labeling plane perpendicular to the A4 segment of the ACAs and ensured that the shimming volume covered the labeling plane, no significant issues arose, provided the region was kept away from the frontal sinus. By obtaining an additional field map at the labeling plane, we could measure the off‐resonance frequency for each vessel^26^. Incorporating this information into the encoding design process could ensure robust vessel encodings,^27^ even when using the optimal settings in VEASL at 7 T^28^.

Moreover, the optimal settings showed a slight improvement in the inversion efficiency at higher velocities. Arteries above the CoW, such as the MCAs, may exhibit blood flow velocities reaching 50 cm/s or even higher.^29^ Conditions such as Moyamoya disease may also lead to a notable increase in flow velocity within the nonstenotic intracranial arteries.^30^ With optimal settings, the impact of the variations in blood flow velocity can be mitigated to a certain extent. In conditions such as Moyamoya disease, where anterior circulation stenosis and occlusion are prevalent, vessel labeling above the CoW can be simplified. The primary focus shifts to the blood supply from posterior cerebral arteries, external arteries, dural arteries, and other collateral pathways rather than labeling multiple branches of MCA. As a result, fewer encoding cycles can be used, reducing scan times (Figure S10), and the labeling plane can be more precisely placed to target the relevant vascular territories.

This study has several limitations. First, simulations modeled only straight arteries, neglecting vessel tortuosity, which could potentially optimize IOES encoding. However, incorporating 3D vessel shapes would significantly increase computational complexity, making it impractical for in vivo scans. We simplified the approach by reducing the labeling plane thickness, accepting some mismatch between expected and actual encodings, which was acceptable for vascular territory mapping but may affect quantitative flow measurements. Moreover, both OES and IOES used Fourier‐based encoding for speed and simplicity, which should be less accurate than designs incorporating true spatial modulation, although the IOES method did use this during the calculation of cost function. Such an approach could be considered in future work, although it is likely this will significantly increase the computational time required. A second limitation was our focus on optimizing labeling efficiency and labeling plane thickness in PCASL without integrating them with encoding designs to improve VEASL SNR efficiency. Combining both could theoretically optimize SNR, but it would require prior knowledge of vessel coordinates, adding substantial computational complexity.

The vessel‐decoding performance also depends on the selection of the labeling plane, which requires an experienced operator. As shown in Figure 5B, the IOES/optimal parameter combination does not guarantee the separation of all vascular territories. This is primarily due to it being challenging to ensure, for each case, that in planes where the PCAs are well separated, all six branches of the MCAs can still be clearly identified, and there is significant individual variability in the branching patterns of the MCA. This issue can be partly mitigated by superselective ASL^5^ because it does not necessitate the same labeling plane for all target arteries. However, this approach can only label one vessel per scan. For multiple vessels, the SNR efficiency is expected to be much lower—and the scan time much longer to achieve image quality comparable to VEASL. Random encoding is an alternative approach, which does not require planning of the encoding patterns.^12^ For the arteries above the CoW, this method typically necessitates a larger number of encodings, around 60, significantly extending the scan time, particularly when each encoding takes some time to acquire, as is the case for angiography. The prolonged duration could then make this impractical for clinical applications. Automated selection of an optimal labeling plane may be a future research topic, potentially explored through deep learning to determine the best labeling plane position, although its feasibility needs further consideration.

## 5 CONCLUSION

This work advances our progress toward enhancing the robustness of vessel‐encoded ASL above the CoW by integrating a more SNR efficient encoding approach and achieving thinner labeling plane, but it is still a challenging problem, particularly with regard to selecting the labeling plane.

## FUNDING INFORMATION

The Wellcome Centre for Integrative Neuroimaging (WIN) is supported by core funding from the Wellcome Trust (203139/Z/16/Z) with additional support from the National Institute for Health and Care Research (NIHR) Oxford Health Biomedical Research Centre (NIHR203316). j.y. is supported by National Natural Science Foundation of China (62401535). z.c. is supported by Natural Science Foundation of Shanghai (22ZR1403900). h.w. is supported by National Key R&D Program of China (2023YFF1204804). t.o. is supported by a Sir Henry Dale Fellowship jointly funded by the Wellcome Trust and the Royal Society (220204/Z/20/Z). For the purpose of open access, the author has applied a Creative Commons by Attribution (CC BY) public copyright license to any Author Accepted Manuscript version arising from this submission.

## CONFLICT OF INTEREST STATEMENT

Thomas Okell is a co‐author of a US patent relating to the VEASL Bayesian analysis method used in this work.

## Supporting information


**Figure S1.** Simulated variation of longitudinal magnetization Mz and contrast due to vessel angulation through the labelling plane. These results show that PCASL labeling efficiency is robust to vessel angulations up to about 60°
**Figure S2.** The inversion profile of bipolar VEASL undergoes a relatively gradual transition between label and control states, where the results for ±50 and ±100 Hz are overlapping, but the unipolar approach leads to a very narrow label region based on the optimized PCASL parameters. In the presence of field inhomogeneity, using unipolar VEASL can also lead to shifts in the encoding pattern.
**Figure S3.** The inversion profile curves measured from two volunteers. When using the bipolar approach, there was not a significant difference in the inversion profile between the default and optimal parameters. Minor deviations between the in vivo results and simulated inversion profile were observed, perhaps due to off‐resonance or scanner drift, but the general trends were consistent. The red and blue bands on the right figure represented the label and control centers in the spatial modulation of VEASL. Initially, the label and control centers were positioned at the RICA and LICA, indicated by the light colors, and were then shifted to the right.
**Figure S4.** (A) The spatial modulation of inversion efficiency for three different RF intervals with optimal settings under three different off‐resonance conditions. (B) Schematic diagram of the SNR efficiency simulation. Three different off‐resonance conditions for the ACAs were considered. (C) Theoretical SNR efficiency results under three different RF intervals and off‐resonance conditions, along with comparisons to OES/default. The first column represents an RF interval of 1560 μs, the middle column represents 1380 μs, and the last column represents 1200 μs.
**Figure S5.** Example VEASL angiographic data in two subjects where the two ACAs were separated within the encoding process. (A) This subject exhibited decoding errors for the LMCA and RPCA (orange arrow) with OES using default setting, with the RACA showing the weakest signal (red arrow), indicating a lower SNR. With IOES, the decoding results were similar regardless of whether the default or optimal parameters were applied. (B) Decoding was comparable for all three methods. However, with OES using the default setting, the signal from the RPCA and LMCA‐1 were weak, indicating low SNR. Additionally, using the default parameters, a slight perturbation of labeling on static tissue was observed (white arrow).
**Figure S6.** Additional datasets: vascular territory maps for three healthy volunteers under the three combinations. (A) In this subject, when using the default PCASL settings, the MCA territory was contaminated by PCA signal for both the OES and IOES cases, which was clearly anatomically incorrect, but this was resolved using the optimized PCASL settings. (B) The territories of both ACAs were not well delineated when using the OES with default setting. (C) The in‐vivo results showed good decoding performance in all three combinations, suggesting that the planning of the labeling plane was optimal and that the subject experienced minimal motion during the scan.
**Figure S7.** Vessel‐encoded angiograms as shown in Figure 8, but this time including the extracranial arteries in the analysis. (A) Even with the inclusion of extracranial vessels, the optimal and IOES combination maintained accurate separation of intracranial arteries while identifying extracranial vessels. (B) In this subject the differences between the two combinations were minimal, and all extracranial arteries have been correctly separated.
**Figure S8.** Vessel‐encoded angiograms for two subjects with some motion during scanning. (A) During both scans, there was considerable head motion from this subject. However, with the optimal and IOES combination, certain intracranial arteries could still be separated even when extracranial vessels were included in the analysis. (B) There was minor head motion from this subject, with two ACAs well separated at the labeling plane, which were included into the encoding design. Both methods decoded most vessels. However, in the default setting, the thickness of the labeling plane might have interfered more with tissue signals, affecting the final visualization.
**Figure S9.** Experimental comparison of RF duty cycle effects on vessel‐encoding performance: (A) In this subject, ten arterial branches (including the left and right ACAs, which were well separated at the labeling plane) were included into the encoding design. Using the optimal setting for IOES with a duty cycle of 63% (right column) led to decoding failures (white arrows) in some MCA territories. (B) Data in another subject, where the clearest boundaries of the perfusion territories were observed only in the left column, with the optimal setting and a duty cycle close to 50%.
**Figure S10.** Comparison of decoding performance using different averages in two healthy volunteers (first two columns) and one Moyamoya patient (last column, the same patient in Figure 2) with IOES and optimal setting. In this Moyamoya patient with LMCA stenosis and occlusion, only 6 vessels were targeted during the encoding process, meaning that only one branch of the LMCA was observed. Therefore, the encoding matrix was derived from an 8‐dimensional Hadamard matrix, plus a non‐selective label, resulting in 9 cycles. For all three subjects, using fewer averages (6 instead of 8) resulted in similar decoding performance, albeit with a slightly lower SNR.
**Figure S11.** Comparison of static B_0_ shimming approaches using the ACAs signal from VEASL angiographic data in one subject. While the optimized PCASL parameters are more sensitive to field inhomogeneity due to the longer RF interval, the shimming region, regardless of whether it included the labeling plane (‘Tag’), minimally influenced the efficacy of vessel‐decoding in the ACAs, as shown by the signal within the ACAs closely matching the simulated signal in all three scenarios. However, there was some apparent signal reduction in the right ACA when the static B_0_ shimming region did not include the labeling plane, so this strategy was avoided in other in vivo experiments.
**Table S1.** Sequence parameters for VTI and angiography using VEASL.

## Data Availability

The code for the IOES calculation and SNR efficiency comparison can be found at https://github.com/SpinEvo/labeling_above_cow. Data underlying some plots in this paper are available at https://zenodo.org/records/14961407.
