# Supplementary material for: Achieving robust labeling above the circle of Willis with vessel‐encoded arterial spin labeling
Source: Magn Reson Med. 2025 Jul 1;94(4):1415–31. doi: 10.1002/mrm.30542 (PMC12309872; doi:10.1002/mrm.30542)
Supplement: Supplementary file 1 — Figure S1. Simulated variation of longitudinal magnetization Mz and contrast due to vessel angulation through the labelling plane. These results show that PCASL labeling efficiency is robust to vessel angulations up to about 60° Figure S2. The inversion profile of bipolar VEASL undergoes a relatively gradual transition between label and control states, where the results for ±50 and ±100 Hz are overlapping, but the unipolar approach leads to a very narrow label region based on the optimized PCASL parameters. In the presence of field inhomogeneity, using unipolar VEASL can also lead to shifts in the encoding pattern. Figure S3. The inversion profile curves measured from two volunteers. When using the bipolar approach, there was not a significant difference in the inversion profile between the default and optimal parameters. Minor deviations between the in vivo results and simulated inversion profile were observed, perhaps due to off‐resonance or scanner drift, but the general trends were consistent. The red and blue bands on the right figure represented the label and control centers in the spatial modulation of VEASL. Initially, the label and control centers were positioned at the RICA and LICA, indicated by the light colors, and were then shifted to the right. Figure S4. (A) The spatial modulation of inversion efficiency for three different RF intervals with optimal settings under three different off‐resonance conditions. (B) Schematic diagram of the SNR efficiency simulation. Three different off‐resonance conditions for the ACAs were considered. (C) Theoretical SNR efficiency results under three different RF intervals and off‐resonance conditions, along with comparisons to OES/default. The first column represents an RF interval of 1560 μs, the middle column represents 1380 μs, and the last column represents 1200 μs. Figure S5. Example VEASL angiographic data in two subjects where the two ACAs were separated within the encoding process. (A) This subject exhibited dec [file MRM-94-1415-s001.docx]

**Supporting information**

**Table S1** Sequence parameters for VTI and angiography using VEASL.

**
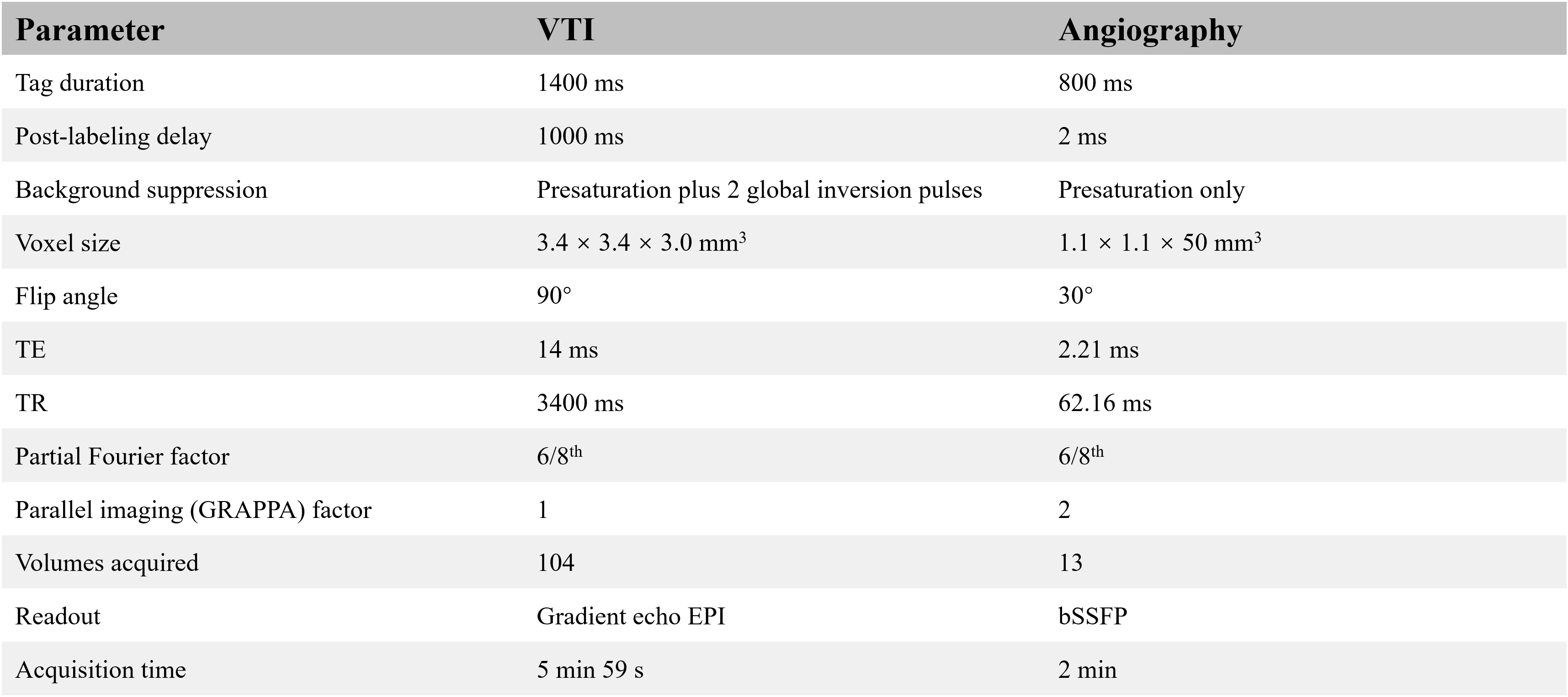
**Abbreviations: VEASL, vessel-encoded arterial spin labeling; VTI, vascular territory imaging


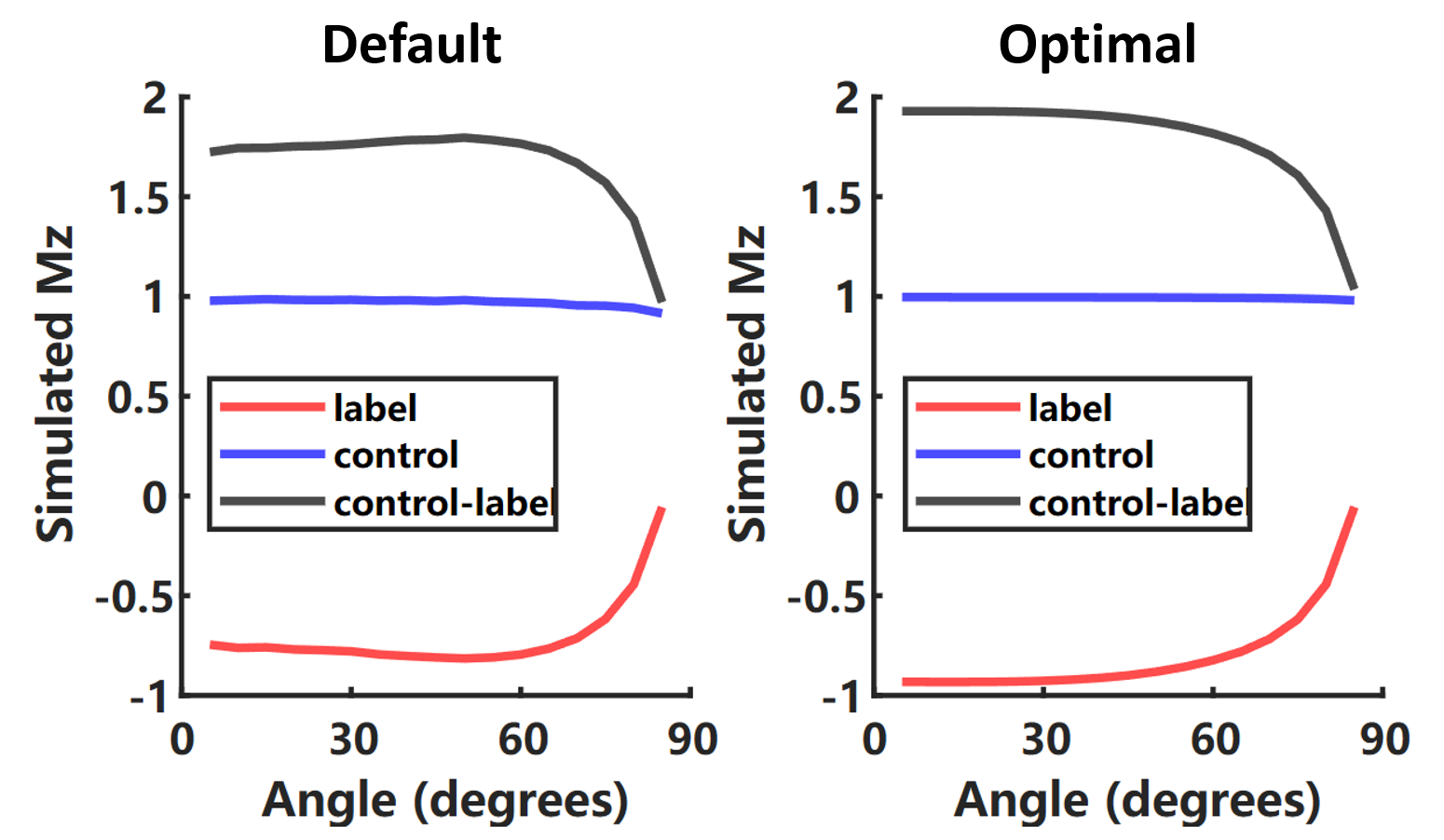


**Figure S1** Simulated variation of longitudinal magnetization Mz and contrast due to vessel angulation through the labelling plane. These results show that PCASL labeling efficiency is robust to vessel angulations up to about 60°

**
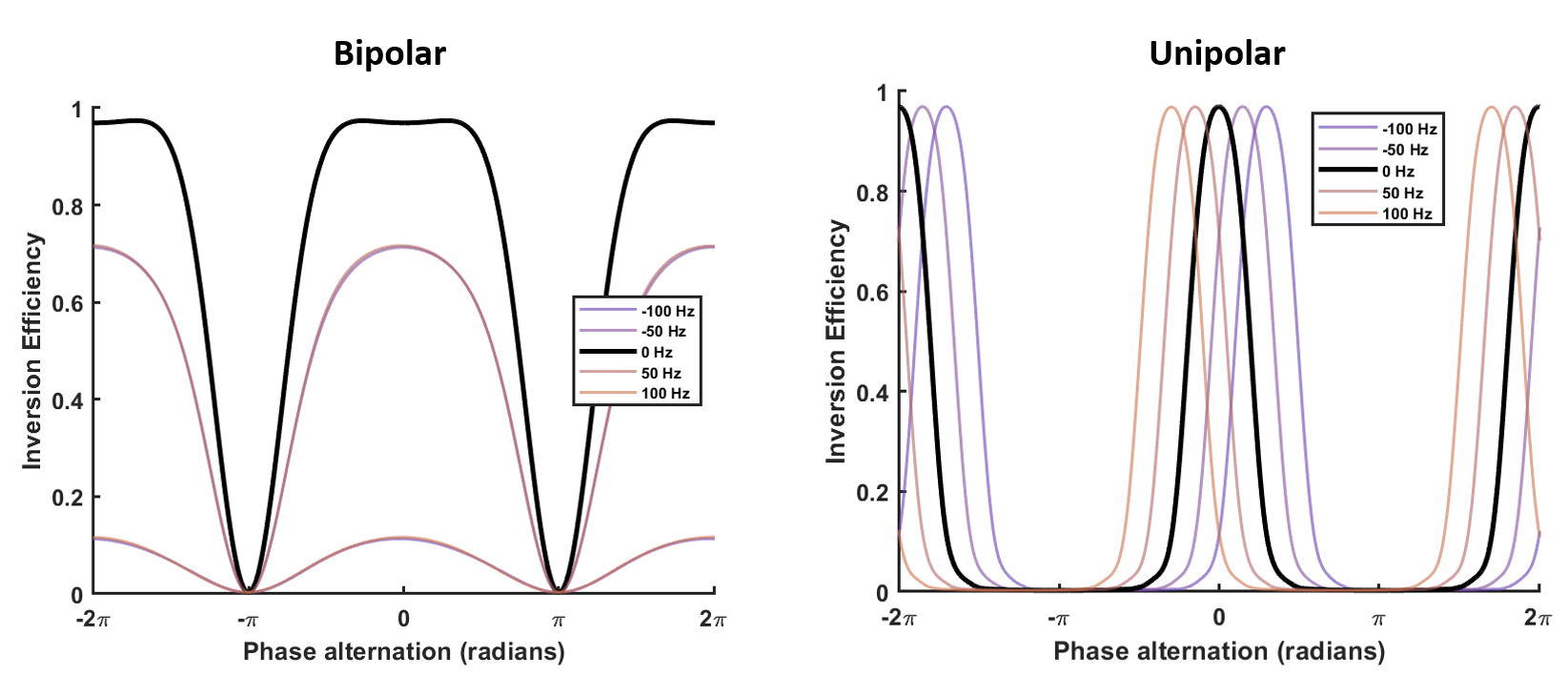
**

**Figure S2** The inversion profile of bipolar VEASL undergoes a relatively gradual transition between label and control states, where the results for ±50 and ±100 Hz are overlapping, but the unipolar approach leads to a very narrow label region based on the optimized PCASL parameters. In the presence of field inhomogeneity, using unipolar VEASL can also lead to shifts in the encoding pattern.

**
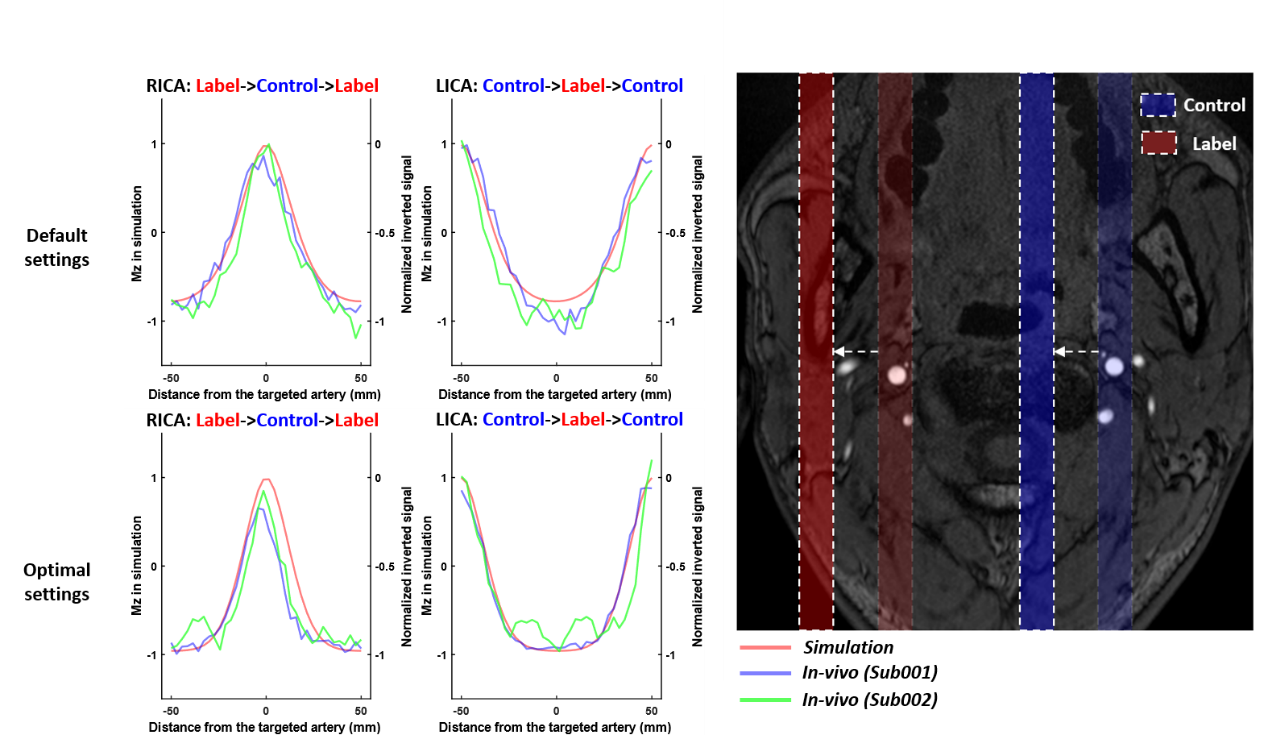
**

**Figure S3** The inversion profile curves measured from two volunteers. When using the bipolar approach, there was not a significant difference in the inversion profile between the default and optimal parameters. Minor deviations between the in vivo results and simulated inversion profile were observed, perhaps due to off-resonance or scanner drift, but the general trends were consistent. The red and blue bands on the right figure represented the label and control centers in the spatial modulation of VEASL. Initially, the label and control centers were positioned at the RICA and LICA, indicated by the light colors, and were then shifted to the right.


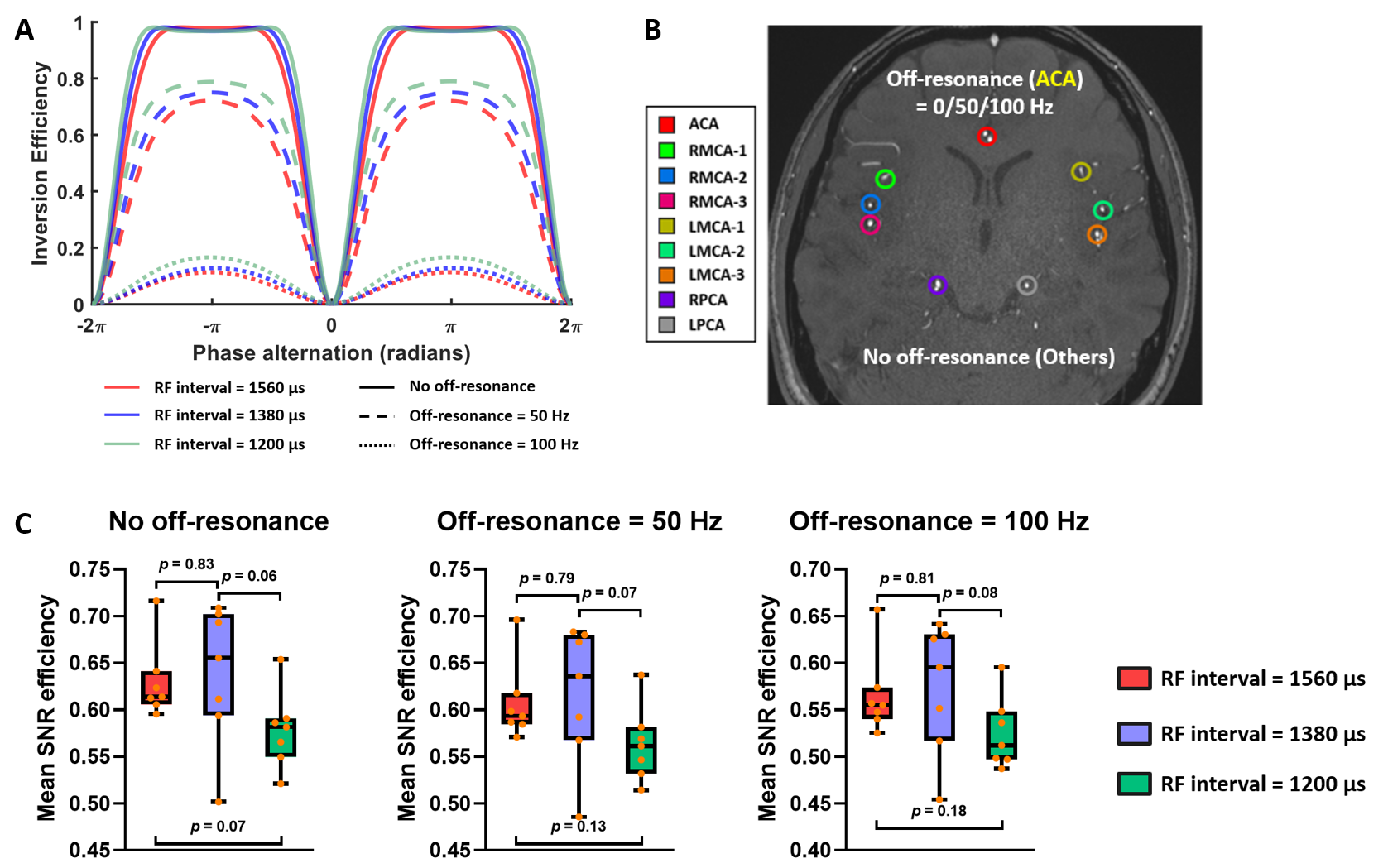
**Figure S4** (A) The spatial modulation of inversion efficiency for three different RF intervals with optimal settings under three different off-resonance conditions. (B) Schematic diagram of the SNR efficiency simulation. Three different off-resonance conditions for the ACAs were considered. (C) Theoretical SNR efficiency results under three different RF intervals and off-resonance conditions, along with comparisons to OES/default. The first column represents an RF interval of 1560 μs, the middle column represents 1380 μs, and the last column represents 1200 μs.


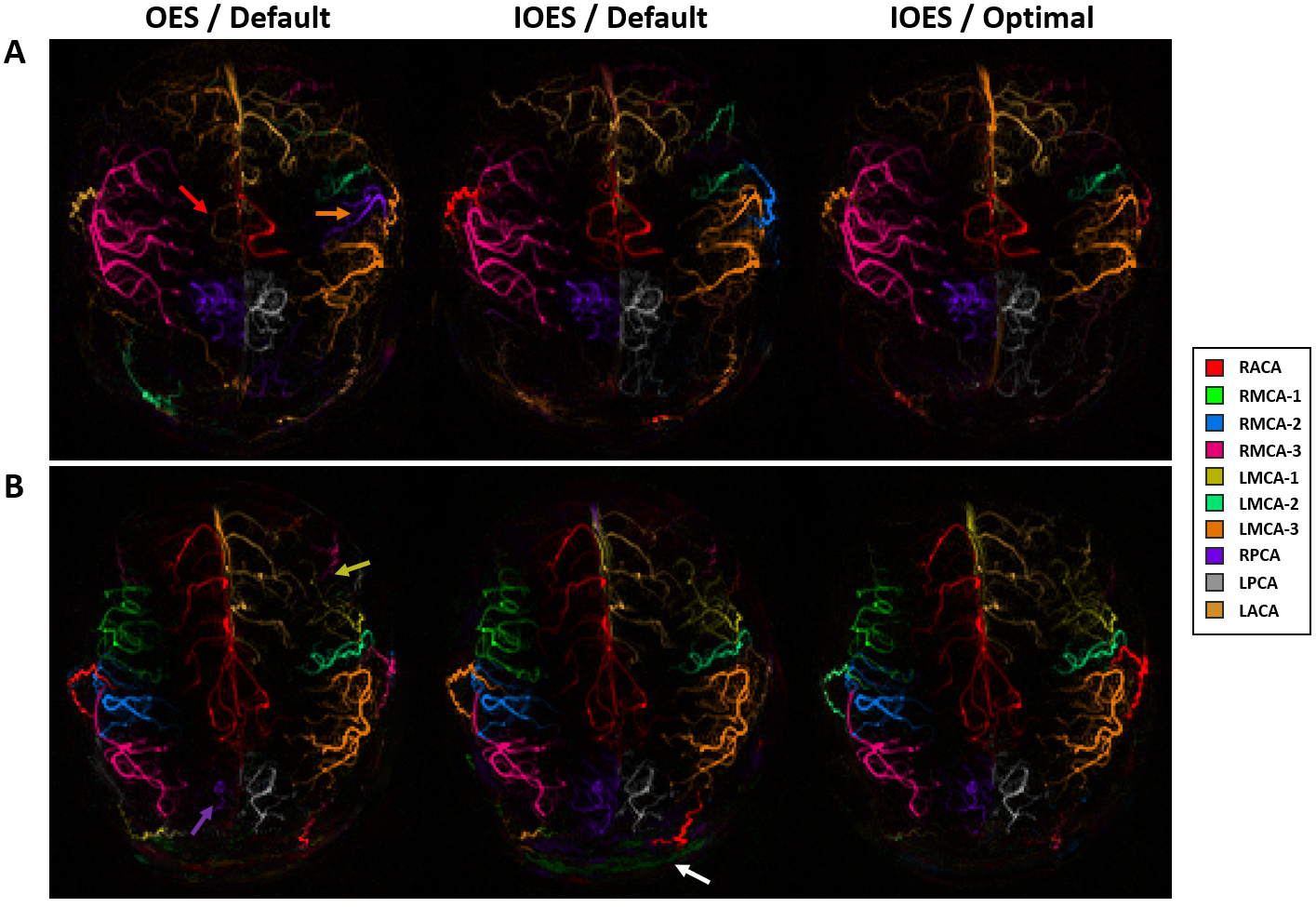


**Figure S5** Example VEASL angiographic data in two subjects where the two ACAs were separated within the encoding process. (A) This subject exhibited decoding errors for the LMCA and RPCA (orange arrow) with OES using default setting, with the RACA showing the weakest signal (red arrow), indicating a lower SNR. With IOES, the decoding results were similar regardless of whether the default or optimal parameters were applied. (B) Decoding was comparable for all three methods. However, with OES using the default setting, the signal from the RPCA and LMCA-1 were weak, indicating low SNR. Additionally, using the default parameters, a slight perturbation of labeling on static tissue was observed (white arrow).


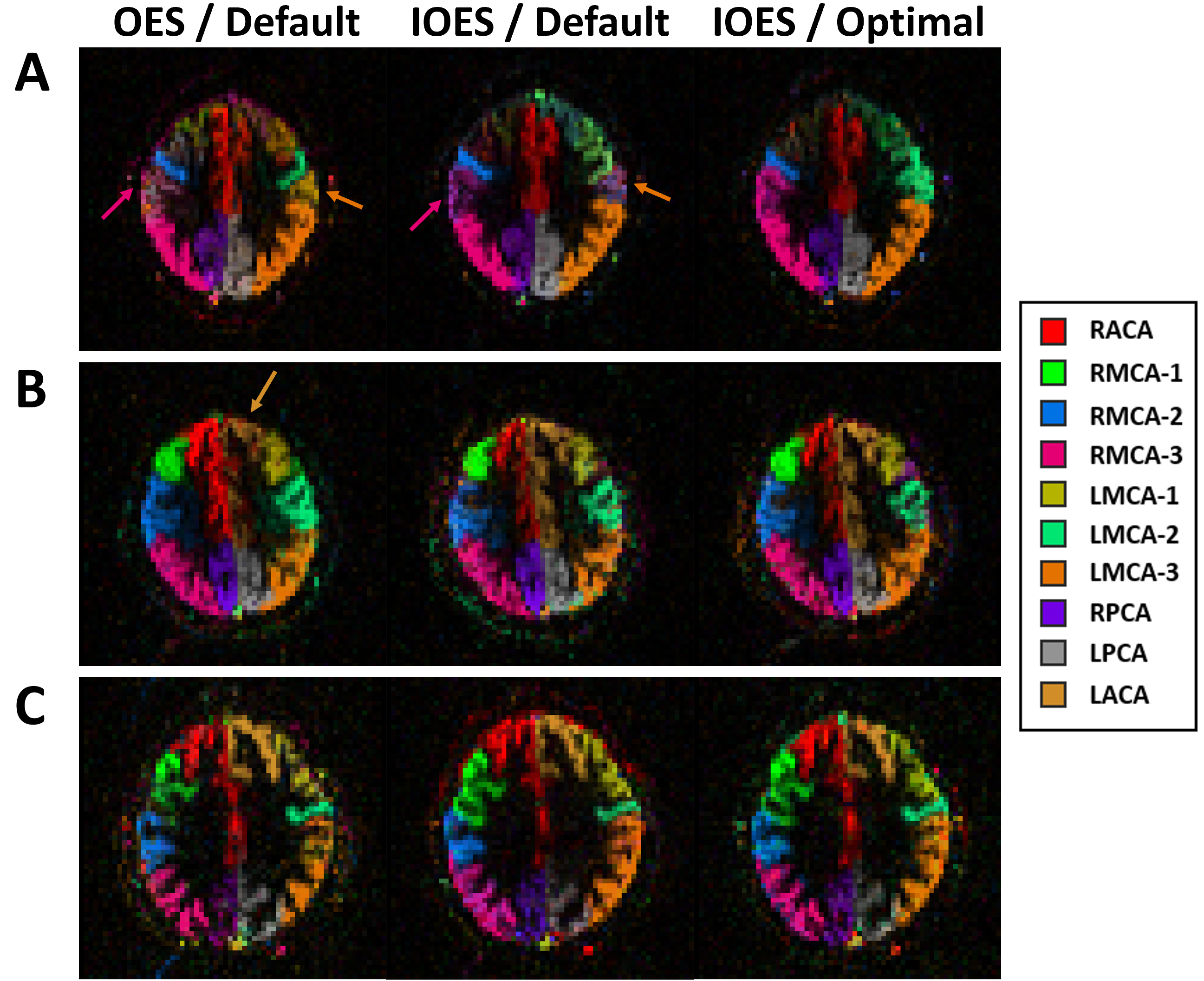


**Figure S6** Additional datasets: vascular territory maps for three healthy volunteers under the three combinations. (A) In this subject, when using the default PCASL settings, the MCA territory was contaminated by PCA signal for both the OES and IOES cases, which was clearly anatomically incorrect, but this was resolved using the optimized PCASL settings. (B) The territories of both ACAs were not well delineated when using the OES with default setting. (C) The in-vivo results showed good decoding performance in all three combinations, suggesting that the planning of the labeling plane was optimal and that the subject experienced minimal motion during the scan.

**
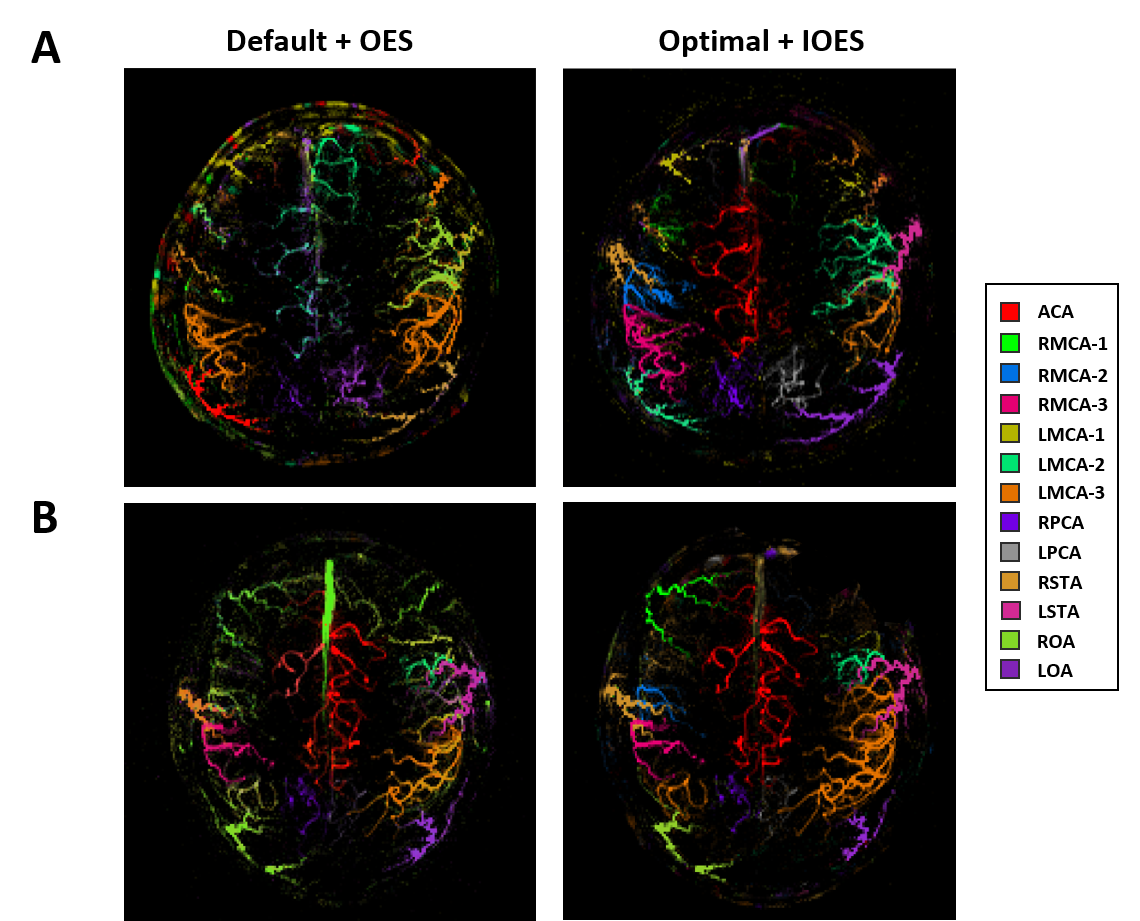
**

**Figure S7** Vessel-encoded angiograms as shown in Figure 6, but this time including the extracranial arteries in the analysis. (A) Even with the inclusion of extracranial vessels, the optimal and IOES combination maintained accurate separation of intracranial arteries while identifying extracranial vessels. (B) In this subject the differences between the two combinations were minimal, and all extracranial arteries have been correctly separated.

**
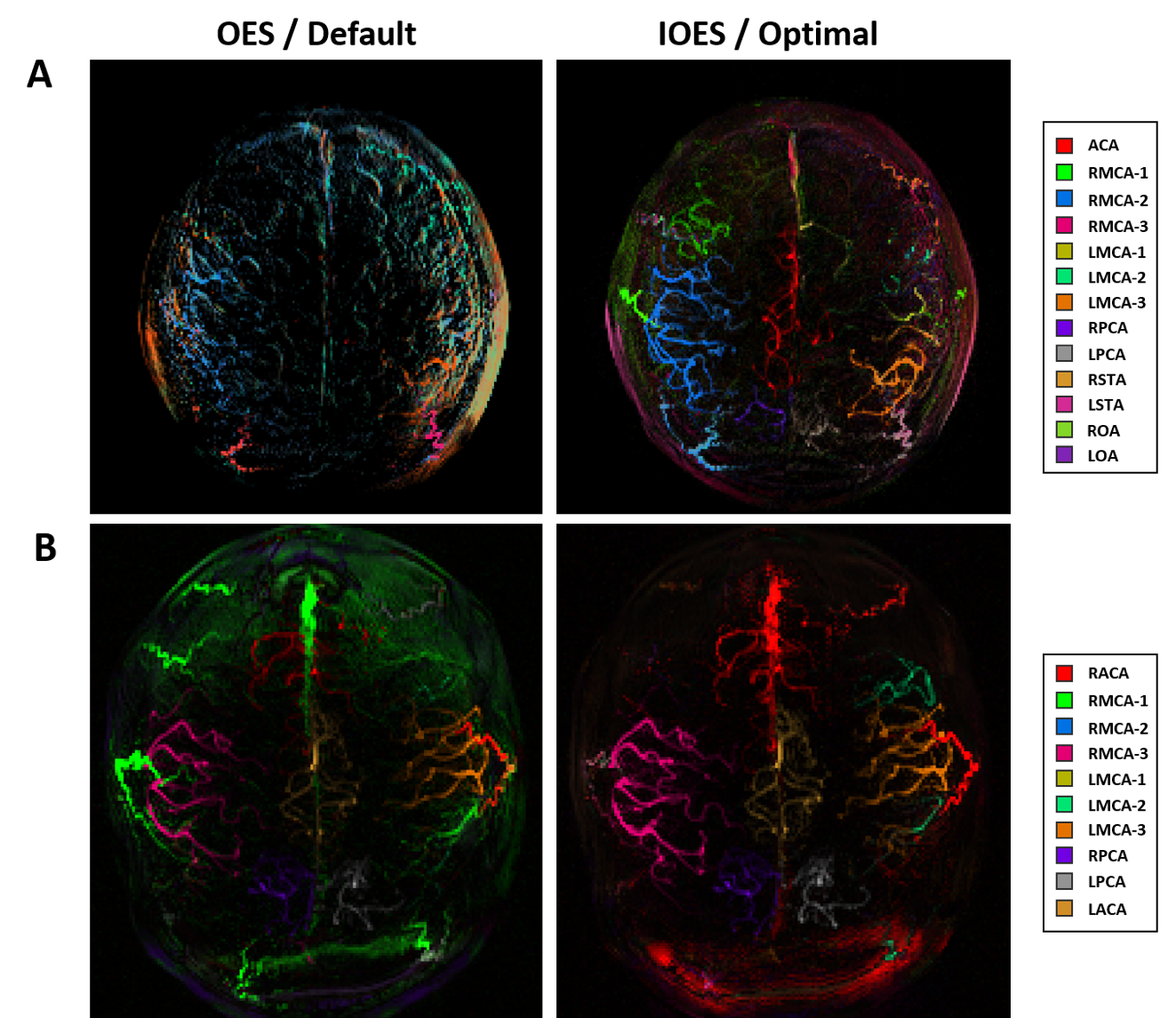
**

**Figure S8** Vessel-encoded angiograms for two subjects with some motion during scanning. (A) During both scans, there was considerable head motion from this subject. However, with the optimal and IOES combination, certain intracranial arteries could still be separated even when extracranial vessels were included in the analysis. (B) There was minor head motion from this subject, with two ACAs well separated at the labeling plane, which were included into the encoding design. Both methods decoded most vessels. However, in the default setting, the thickness of the labeling plane might have interfered more with tissue signals, affecting the final visualization.


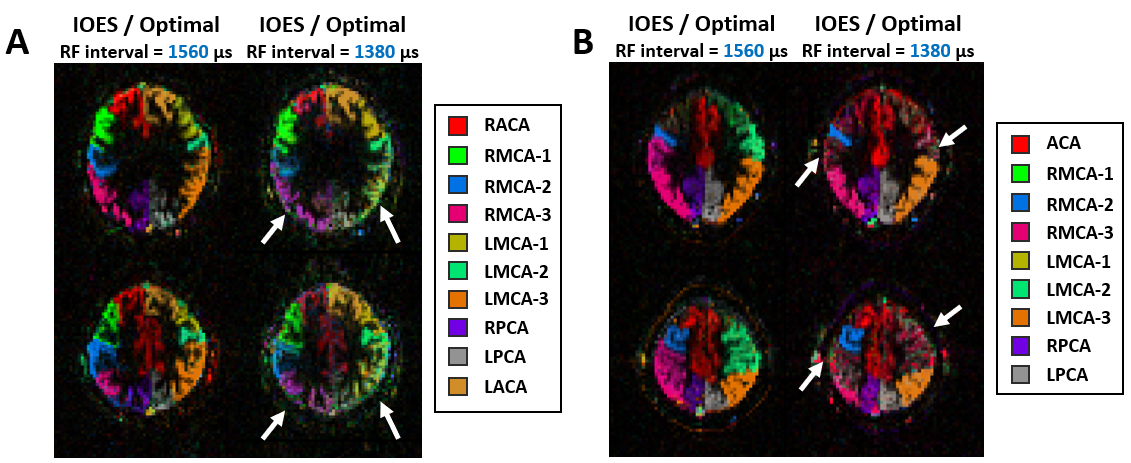


**Figure S9** Experimental comparison of RF duty cycle effects on vessel-encoding performance: (A) In this subject, ten arterial branches (including the left and right ACAs, which were well separated at the labeling plane) were included into the encoding design. Using the optimal setting for IOES with a duty cycle of 63% (right column) led to decoding failures (white arrows) in some MCA territories. (B) Data in another subject, where the clearest boundaries of the perfusion territories were observed only in the left column, with the optimal setting and a duty cycle close to 50%.


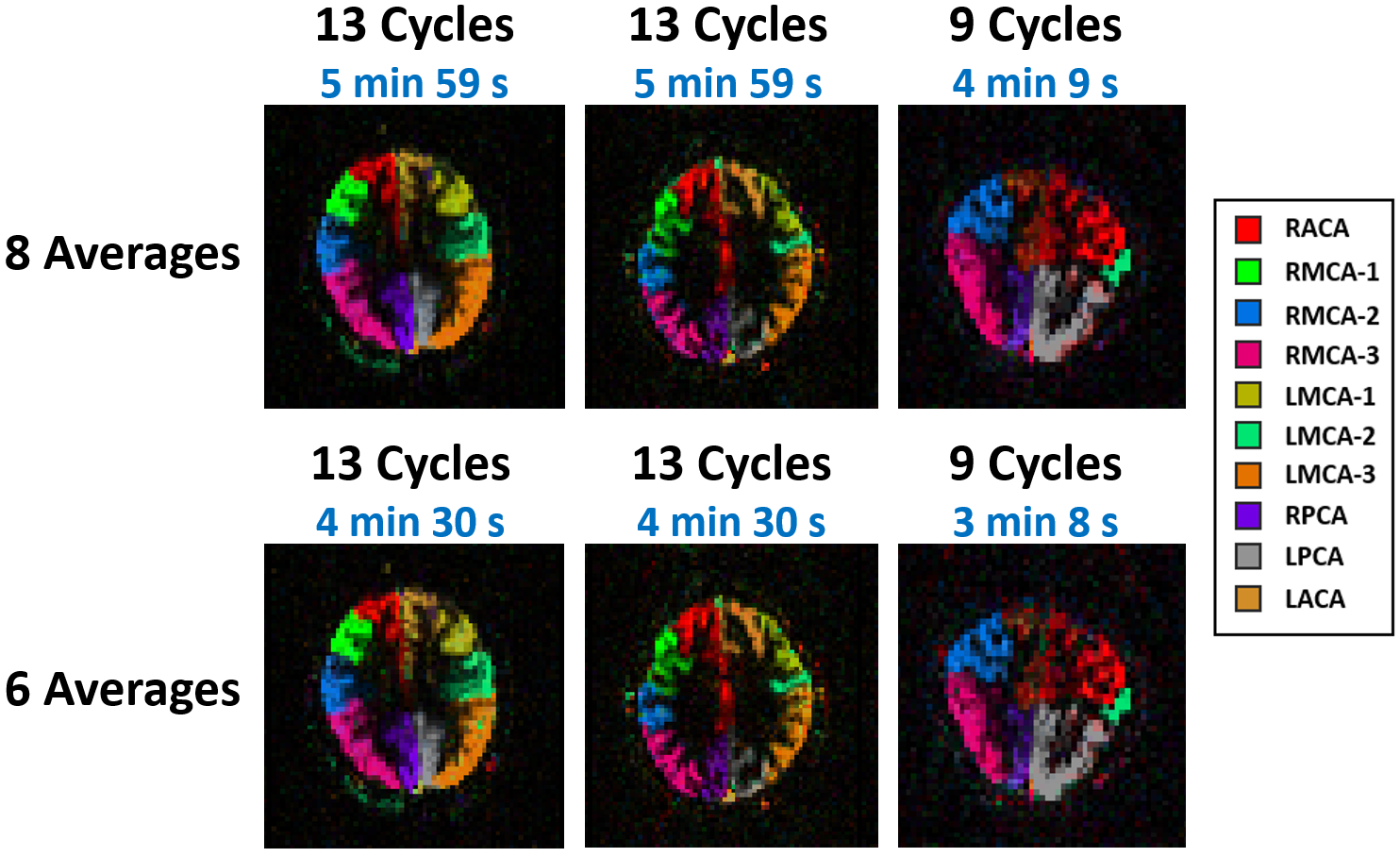


**Figure S10** Comparison of decoding performance using different averages in two healthy volunteers (first two columns) and one Moyamoya patient (last column, the same patient in **Figure 8**) with IOES and optimal setting. In this Moyamoya patient with LMCA stenosis and occlusion, only 6 vessels were targeted during the encoding process, meaning that only one branch of the LMCA was observed. Therefore, the encoding matrix was derived from an 8-dimensional Hadamard matrix, plus a non-selective label, resulting in 9 cycles. For all three subjects, using fewer averages (6 instead of 8) resulted in similar decoding performance, albeit with a slightly lower SNR.

**
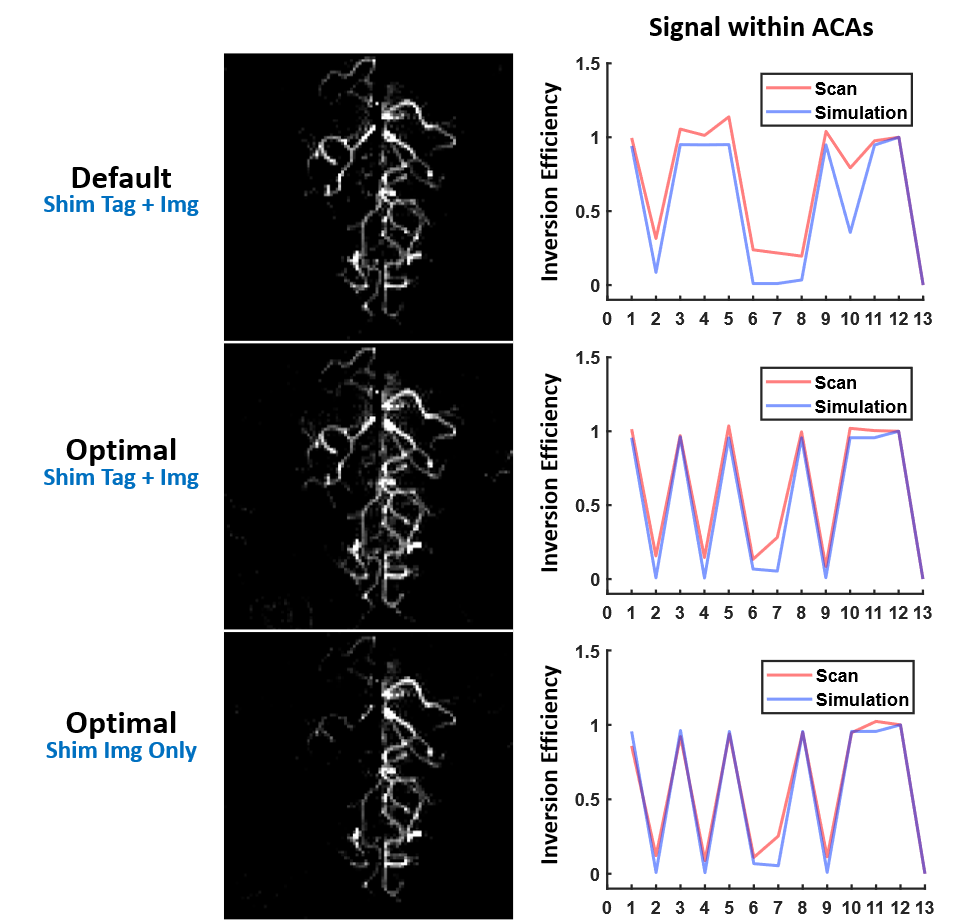
**

**Figure S11:** Comparison of static B_0_ shimming approaches using the ACAs signal from VEASL angiographic data in one subject. While the optimized PCASL parameters are more sensitive to field inhomogeneity due to the longer RF interval, the shimming region, regardless of whether it included the labeling plane ('Tag'), minimally influenced the efficacy of vessel-decoding in the ACAs, as shown by the signal within the ACAs closely matching the simulated signal in all three scenarios. However, there was some apparent signal reduction in the right ACA when the static B_0_ shimming region did not include the labeling plane, so this strategy was avoided in other in vivo experiments.
